# Thermoregulation of functional amyloid Fap-dependent biofilm formation via cyclic diguanosine monophosphate signaling in *Pseudomonas fluorescens*

**DOI:** 10.1128/aem.02387-25

**Published:** 2026-03-20

**Authors:** Yinan Ru, Siqi Tan, Xinyi Shen, Chongni Zheng, Yinying Wu, Yunhai Shao, Xiaoxiang Liu

**Affiliations:** 1 School of Basic Medical Sciences and Forensic Medicine, Hangzhou Medical College 117839, Hangzhou, Zhejiang, China; 2 School of Public Health, Hangzhou Medical College 117839, Hangzhou, Zhejiang, China; 3 Nanxun District Center for Disease Control and Prevention, Huzhou, Zhejiang, China; Anses, Maisons-Alfort Laboratory for Food Safety, Maisons-Alfort, France

**Keywords:** temperature, biofilm, c-di-GMP, functional amyloid Fap, *Pseudomonas fluorescens*

## Abstract

We recently reported that *Pseudomonas fluorescens* PF07, an isolate from refrigerated marine fish, produces the functional amyloid Fap as the major component of its biofilm matrix and that transcription of the *fap* gene cluster is directly regulated by BrfA, a novel c-di-GMP-responsive transcription regulator. As a psychrotrophic food spoiler, *P. fluorescens* encounters temperature fluctuations during food processing and distribution; however, the effects of temperature on its biofilm formation remain poorly characterized. Here, we show that reduced temperatures (4°C and 15°C) significantly attenuate macrocolony, pellicle, and solid-surface-associated biofilm formation in PF07 compared to 28°C. Mechanistically, low temperatures suppress Fap-dependent biofilm formation by downregulating intracellular c-di-GMP levels via the coordinated control of two key enzymes: a novel diguanylate cyclase, DebA, and a cold-adapted phosphodiesterase BifA. At 28°C, DebA is highly expressed and maintains robust catalytic activity via its PAS domain, while BifA exhibits low activity due to poor thermostability; these effects together drive c-di-GMP accumulation, *fap* expression, and biofilm formation. Conversely, low temperatures reduce DebA expression and activity, while BifA retains exceptional cold tolerance to accelerate c-di-GMP degradation, thereby suppressing *fap* expression and biofilm formation. This study delineates a novel temperature-responsive c-di-GMP signaling pathway in the psychrotrophic food spoiler, *P. fluorescens* PF07.

**IMPORTANCE:**

The persistence of bacteria in various biofilms frequently leads to food spoilage and foodborne illnesses. *Pseudomonas fluorescens* is widely recognized as one of the most prevalent spoilage organisms, with a robust capacity for biofilm formation. Temperature is a critical factor in food processing, distribution, and preservation. This study identifies a novel temperature-responsive c-di-GMP signaling module centered on the novel diguanylate cyclase DebA and the cold-adapted phosphodiesterase BifA, which governs Fap-dependent biofilm formation in *P. fluorescens* PF07. Our findings expand the known repertoire of c-di-GMP-mediated biofilm regulatory pathways and may inform the development of improved antibiofilm strategies for the food industry.

## INTRODUCTION

Food products are susceptible to contamination by spoilage organisms and pathogens through contact between the food matrix and processing equipment during food handling and processing. Bacteria adhering to food or equipment surfaces typically form biofilms, spatially organized sessile structures in which bacterial cells are encased within a self-produced extracellular matrix (ECM) (1, 2). The biofilm matrix generally comprises proteins, exopolysaccharides, and nucleic acids. ECM production leads to a mature and rigid biofilm, which enhances bacterial survival and persistence in the environment and complicates bacterial removal using standard sanitation procedures (3). Bacteria form macrocolony biofilms on solid organic materials (e.g., solid foods), pellicles at the air-liquid interface (e.g., liquid foods), and solid-surface-associated (SSA) biofilms on abiotic substrates (e.g., processing equipment) (4, 5). The persistence of bacteria within these biofilm architectures frequently causes food spoilage, product rejection, economic losses, and foodborne illnesses (6). *Pseudomonas* species are the predominant spoilers of proteinaceous raw foods (e.g., raw milk, meats, fish, and ready-to-eat products) stored under aerobic refrigerated conditions (7, 8). *Pseudomonas fluorescens* is currently recognized as one of the most prevalent spoilage organisms with a high capacity for biofilm formation (9). Despite routine cleaning and sanitation protocols, *P. fluorescens* can persist on the surfaces of processing equipment in its biofilm form (10, 11).

The second messenger cyclic diguanosine monophosphate (c-di-GMP) is an intracellular signaling molecule that regulates biofilm formation, motility, virulence, and other cellular processes (12). In general, high intracellular c-di-GMP levels upregulate ECM component production, enabling bacteria to form biofilms, whereas low c-di-GMP levels downregulate ECM component production, triggering biofilm dispersal into a planktonic growth mode (13). c-di-GMP is synthesized by diguanylate cyclases (DGCs), which contain GGDEF domains, and is degraded by phosphodiesterases (PDEs), which contain EAL or HD-GYP domains (14). Bacteria often harbor multiple DGCs and PDEs, some of which modulate ECM production during biofilm formation by fine-tuning c-di-GMP levels (2, 15). *P. fluorescens* SBW25, an isolate from sugar beet leaves, produces cellulose Wss as a key component of the ECM. Studies have shown that DGCs (e.g., WspR, YfiN/AwsR) and PDEs (e.g., SwsR) regulate Wss production to drive the formation of wrinkled pellicles and macrocolony biofilms (16). In the rhizobacterium *P. fluorescens* strain Pf0-1, the large adhesion protein LapA was identified as an important biofilm matrix component. LapD, an inner membrane c-di-GMP effector protein, inhibits the activity of LapG (a periplasmic cysteine protease), thereby retaining the adhesin LapA on the cell surface under high c-di-GMP conditions (17). A systematic analysis of genes encoding GGDEF and EAL domains in *P. fluorescens* Pf0-1 revealed that two DGCs (GcbB and GcbC) and one PDE (RapA) modulate SSA biofilm formation by controlling LapA localization to the cell surface (15, 18). Our recent work showed that *P. fluorescens* PF07, an isolate from spoiled marine fish stored at 4°C, produces the functional amyloid Fap encoded by the gene cluster *fapA–F* as the major biofilm matrix component (19) and that transcription of *fapA* is directly regulated by a novel c-di-GMP-responsive transcription regulator, BrfA (20). The Fap functional amyloids and BrfA-type transcription factors are widespread across *Pseudomonas* species (20). Functional amyloids are generally resistant to thermal, proteolytic, and chemical denaturation, imparting hydrophobicity and mechanical robustness to biofilms (21, 22). However, the specific DGCs and PDEs that critically regulate intracellular c-di-GMP levels and *fap* expression in PF07 and other *Pseudomonas* strains remain uncharacterized.

Environmental signals can regulate the activity and expression of DGCs and PDEs, thereby affecting biofilm development. For example, in *Pseudomonas aeruginosa*, the activity of the DGC SadC is modulated by oxygen availability (23), and the DGC TdcA directly senses temperature through its thermosensitive Per-Arnt-Sim (PAS) domain (24). Regulation of c-di-GMP-metabolizing enzymes also occurs at the transcriptional level. In *P. putida*, expression of the DGC gene *cfcR* is entirely dependent on the sigma factor RpoS and is positively regulated by the transcription regulator FleQ (25, 26), while the transcription of the PDE gene *bifA* is partially controlled by the flagellar sigma factor FliA (27). *P. fluorescens* is a psychrotrophic bacterium widely distributed in natural habitats. In food systems, it encounters temperature changes during processing and distribution; however, little is known about the effects of temperature on its biofilm formation. In this study, we demonstrate that reduced temperatures inhibit biofilm formation in *P. fluorescens* PF07 by decreasing intracellular c-di-GMP levels and repressing *fap* expression. We further identify a novel DGC, designated DebA for “DGC essential for biofilm formation,” and the cold-adapted PDE BifA as key mediators of the temperature-dependent regulation of Fap-dependent biofilm formation.

## RESULTS

### Low temperatures inhibit the formation of red and wrinkled macrocolony biofilms and the production of biofilm matrices

**Fig 1:**
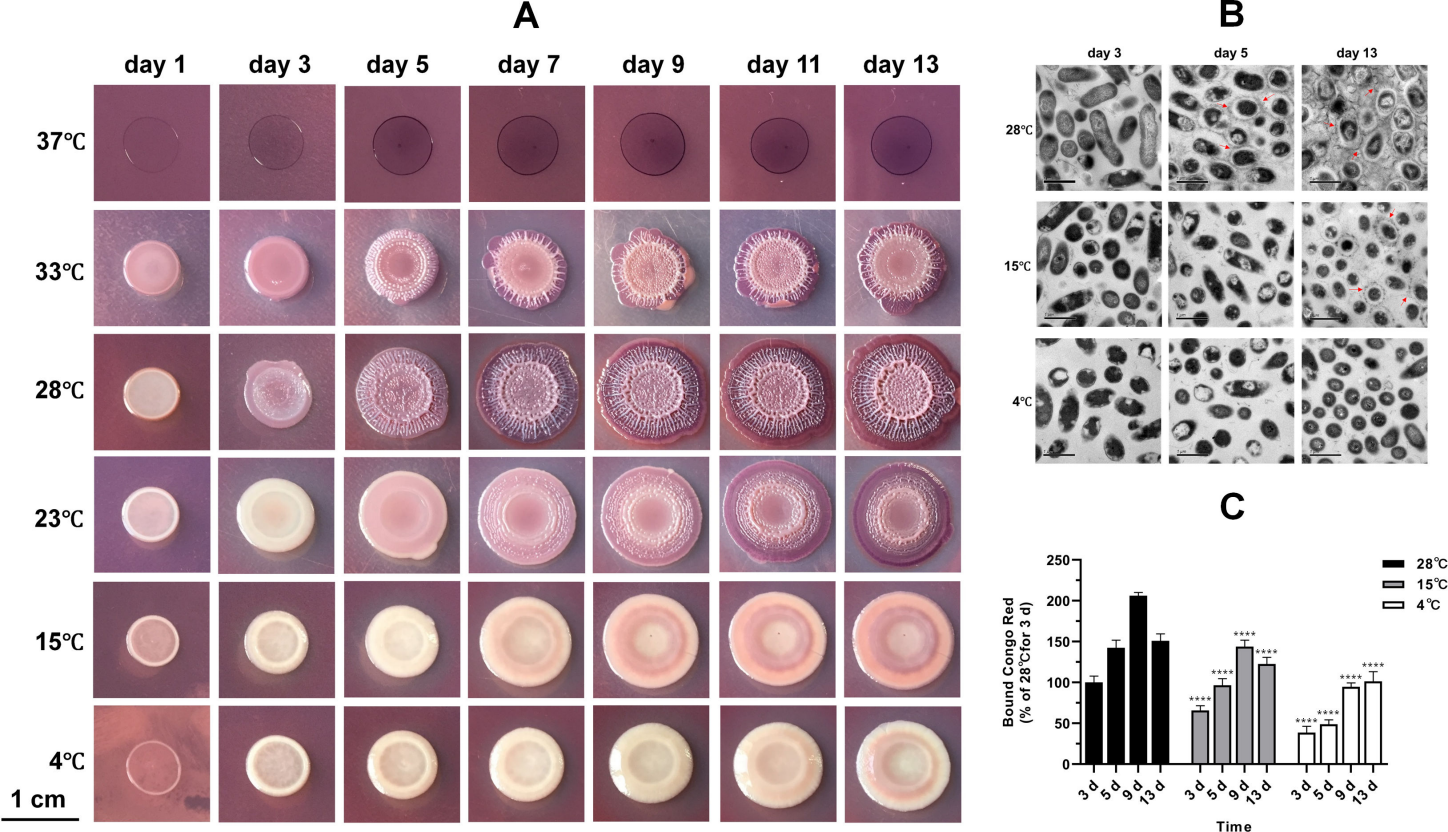
Low temperatures inhibit macrocolony biofilm formation and biofilm matrix production in *P. fluorescens* PF07. (**A**) Macrocolony biofilms formed on Congo red plates (scale bar = 1 cm). The plates were incubated at 4°C, 15°C, 23°C, 28°C, 33°C, or 37°C for 13 days. Representative images from at least six biological replicates are shown. (**B**) Transmission electron micrographs of the macrocolony biofilms (scale bar = 1 µm). Macrocolonies incubated at 4°C, 15°C, and 28°C for 3, 5, and 13 days were gently scraped from tryptone agar plates and observed via transmission electron microscopy (TEM). Representative images are shown, and matrix materials surrounding the cells are marked with red arrows. (**C**) Time-course quantification of extracellular matrix (ECM) in PF07 macrocolonies cultured at different temperatures using the Congo red binding assay. ECM content is presented as a percentage relative to the 28°C 3-day culture sample. Data are presented as mean ± standard deviation (SD) of three biological replicates, each with three technical replicates. Two-way ANOVA followed by Dunnett’s multiple comparisons test was used to determine statistical significance relative to the 28°C group at each time point (^****^P < 0.0001).

The functional amyloid Fap, the major component of the biofilm matrix, drives *P. fluorescens* PF07 to form red and wrinkled macrocolonies on Congo red plates (19). To determine the influence of temperature on the development of PF07 macrocolony biofilms, macrocolony morphology assays were first performed on Congo red plates across a temperature gradient of 4°C to 37°C. Considering the effect of temperature on growth, we extended the observation period to 13 days. As shown in Fig. 1A, PF07 incubated at 28°C developed a red and wrinkled macrocolony biofilm by day 3, with staining intensity and surface roughness increasing progressively as incubation continued. When the temperature was increased to 33°C, the strain formed red and wrinkled macrocolonies, although colony growth was somewhat inhibited. At 37°C, no bacterial growth was observed, likely because this temperature exceeds the upper growth limit of PF07. Conversely, when temperatures were reduced to 23°C, 15°C, and 4°C, the colonies were able to grow, but the Congo red staining and surface roughness decreased markedly with decreasing temperature. Only smooth and pale colonies were observed after incubation at 15°C or 4°C for up to 13 days. Next, macrocolony biofilm structures of PF07 cultured at 28°C, 15°C, and 4°C were visualized using transmission electron microscopy (TEM) (Fig. 1B). At 28°C, visible ECM was produced in the macrocolony biofilms from day 5, whereas only negligible amounts of ECM were observed in colonies cultured at 15°C for 13 days, and no ECM was observed at 4°C even after 13 days of incubation. Finally, ECM production in PF07 colonies was quantified over time at different incubation temperatures using a Congo red binding assay, which revealed that ECM levels at 28°C were significantly higher than those at 15°C and 4°C (Fig. 1C). These results demonstrate that a higher temperature (28°C) promotes macrocolony biofilm formation and biofilm matrix production in PF07, whereas lower temperatures significantly inhibit these processes. Additionally, we examined the effect of temperature on the macrocolony biofilms of another *P. fluorescens* strain, UK4, a Fap-producing isolate from a drinking water reservoir (28). Consistent with PF07, the formation of red and wrinkled macrocolony biofilms by UK4 was also significantly inhibited by low temperatures (Fig. S1).

### Low temperatures inhibit pellicle and SSA biofilm formation

**Fig 2:**
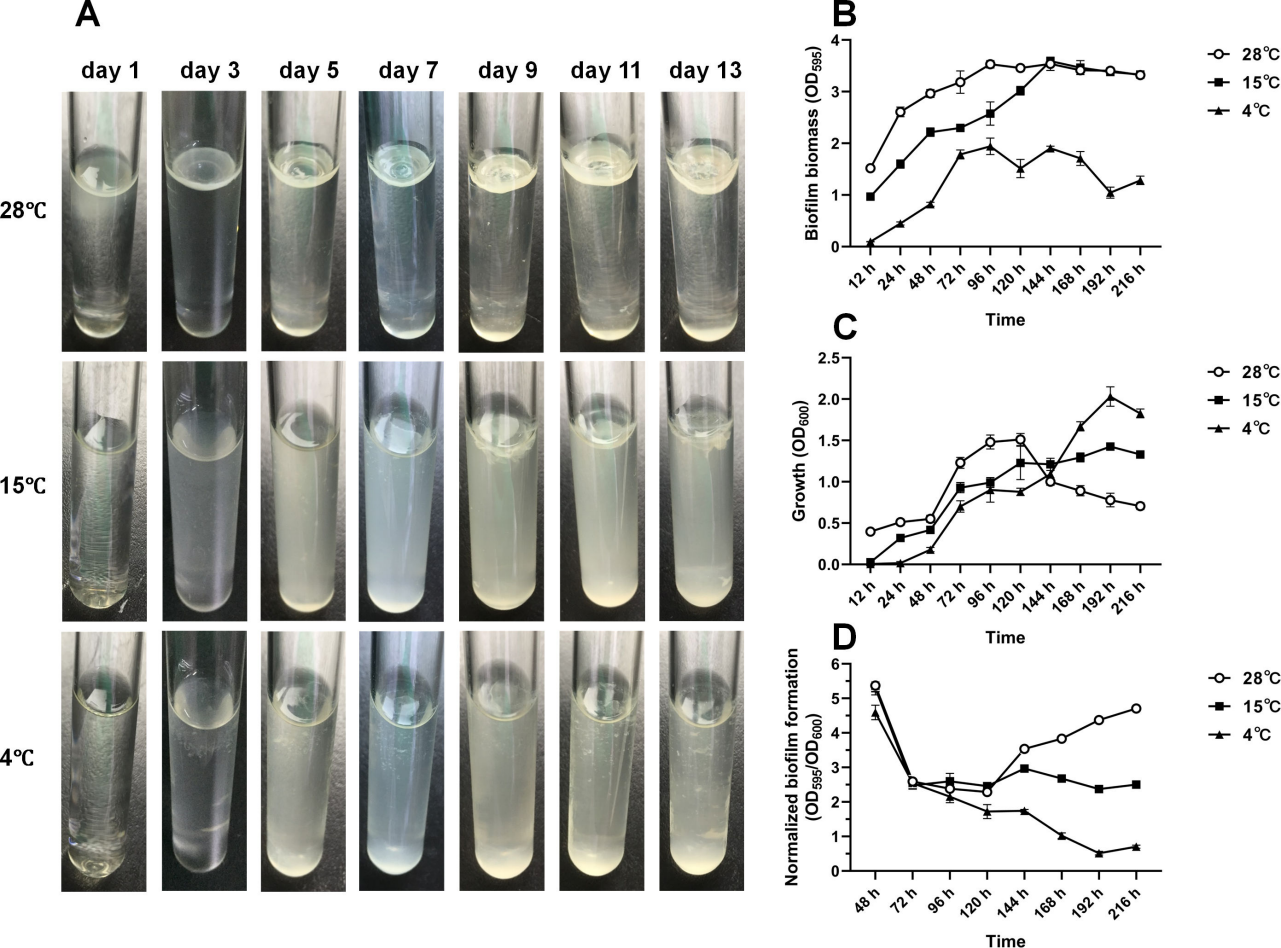
Low temperatures inhibit pellicle and solid-surface-associated (SSA) biofilm formation in *P. fluorescens* PF07. (**A**) PF07 pellicles formed at the air-liquid interface of static cultures. The cultures were statically incubated in glass tubes at 28°C, 15°C, or 4°C for 13 days. The image is representative of at least three biological replicates. (**B–D**) Quantification of SSA biofilms in 24-well microplates under shaking conditions (130 rpm) at 28°C, 15°C, or 4°C for 216 h: (**B**) biofilm biomass quantified by crystal violet staining (OD₅₉₅); (**C**) planktonic growth determined by measuring the OD_600_; and (**D**) normalized biofilm biomass (OD₅₉₅/OD₆₀₀, biofilm biomass normalized to planktonic growth). Data are presented as mean ± SD of eight biological replicates.

PF07 can form robust pellicles at the air-liquid interface of static liquid cultures and SSA biofilms on abiotic solid surfaces (19). Therefore, the effects of temperature on pellicle and SSA biofilm formation were determined in this strain. Initially, PF07 was cultured in static tryptone broth at 28°C, 15°C, and 4°C (Fig. 2A). At 28°C, discernible pellicles emerged on the culture surface from day 3, and these structures progressively thickened and developed increased wrinkling over time. Meanwhile, the liquid medium remained relatively clear throughout the 13-day incubation period. In contrast, no intact pellicles were observed over the entire incubation period at 15°C and 4°C, and the liquid medium became progressively turbid under these low-temperature conditions. PF07 was then inoculated into 2 mL of tryptone broth in 24-well microplates and gently agitated at 28°C, 15°C, and 4°C for 12–216 h. Adherent biofilm biomass and planktonic cell density were subsequently quantified. As shown in Fig. 2B, the biofilm biomass produced at 28°C was significantly higher than that at 15°C and 4°C within the first 120 h of culture. After 120 h, biofilm biomass at 15°C progressively increased, reaching levels comparable to those at 28°C, whereas that at 4°C remained at a relatively low level. As shown in Fig. 2C, the planktonic growth of PF07 was also temperature-dependent. Within the first 120 h of incubation, OD_600_ values at 28°C were significantly higher than those at 15°C and 4°C. After 120 h, the OD_600_ at 28°C decreased, while values at 15°C and 4°C exceeded those at 28°C. Notably, over the entire 216-h incubation period, the maximum OD₆₀₀ value at 4°C exceeded the highest value recorded at 28°C, indicating that incubation at 4°C enables PF07 to achieve higher planktonic cell densities during the stationary phase compared to incubation at 28°C. To eliminate the confounding effect of bacterial cell concentration on biofilm formation, biofilm biomass (quantified via crystal violet staining as OD₅₉₅) was normalized to planktonic density (OD_600_) as described by Han et al. (29). As shown in Fig. 2D, the normalized biofilm formation at 28°C was comparable to that at 15°C and 4°C within the first 120 h but was significantly higher than that at 15°C and 4°C after 120 h. These results demonstrate that low temperatures markedly inhibit pellicle and SSA biofilm formation in PF07.

### Low temperatures decrease *fap* gene expression and intracellular c-di-GMP levels

**Fig 3:**
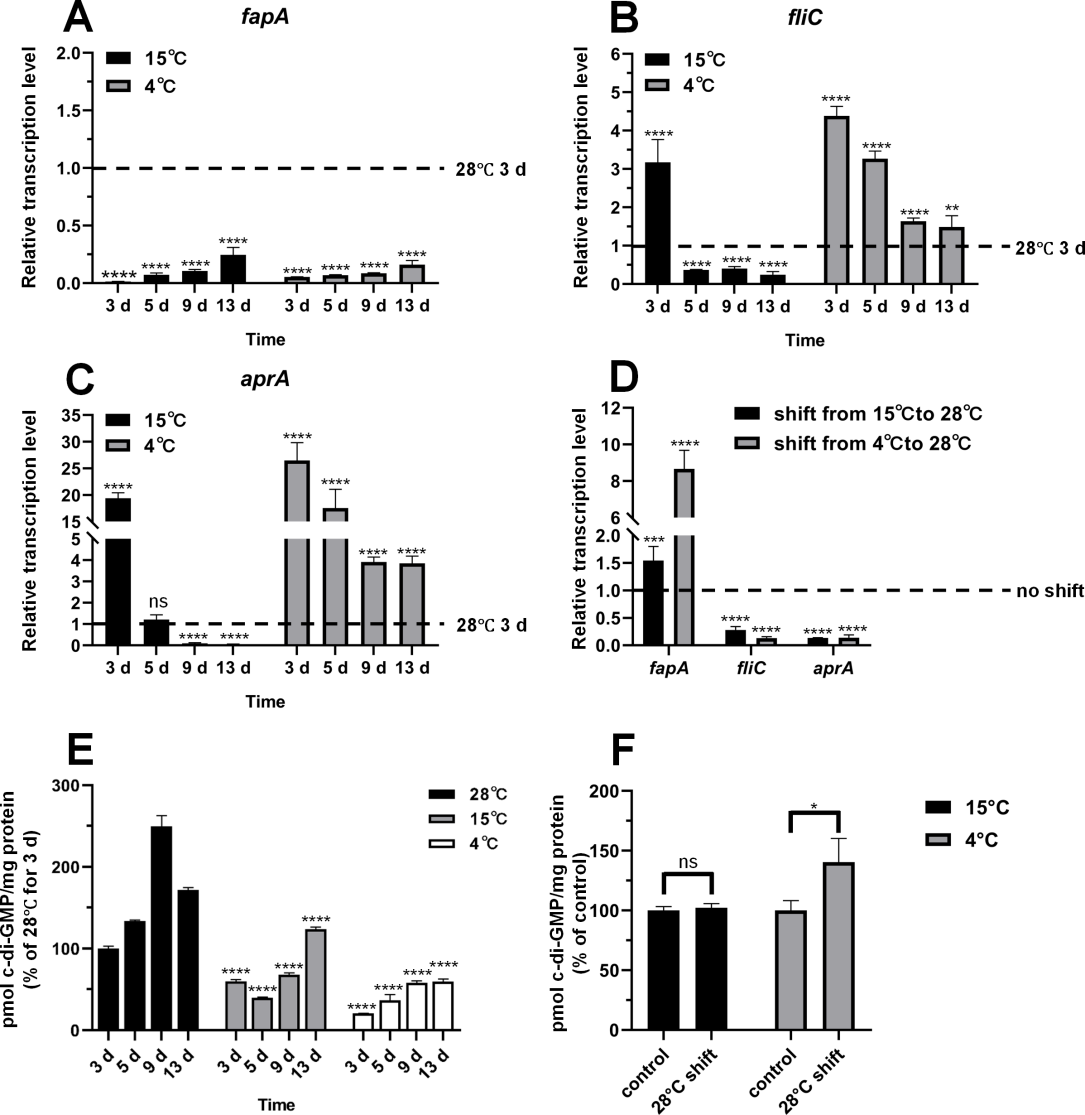
*fapA*, *fliC*, and *aprA* expression and intracellular c-di-GMP levels are thermoregulated in *P. fluorescens* PF07. (**A–C**) Time course of gene expression in PF07 macrocolonies at different temperatures, as determined by qRT-PCR. The expression levels are relative to those at 28°C for 3 days. One-way ANOVA with Dunnett’s T3 multiple comparison test was used to determine statistical significance compared to the levels at 28°C for 3 days. (**D**) Relative expression levels of genes in PF07 macrocolonies after a temperature shift. PF07 colonies were cultured on tryptone plates at 15°C or 4°C for 3 days, followed by a shift to 28°C for 1 day. The expression levels determined by qRT-PCR are relative to those of control cultures maintained without a temperature shift. A two-tailed unpaired *t*-test with Welch’s correction was used to compare expression levels between the samples with and without a temperature shift. The gene expression data are mean ± SD of three biological replicates, each with three technical replicates. (**E**) Time-course quantification of intracellular c-di-GMP levels in PF07 macrocolonies cultured at different temperatures. The c-di-GMP levels are expressed as a percentage relative to those at 28°C for 3 days. Two-way ANOVA followed by Dunnett’s multiple comparisons test was used to determine statistical significance relative to the 28°C group at each time point. (**F**) Intracellular c-di-GMP quantification in PF07 macrocolonies cultured at 15°C or 4°C for 3 days, followed by a shift to 28°C for 1 day. The c-di-GMP levels are expressed as a percentage relative to those of control cultures maintained without a temperature shift. A two-tailed unpaired *t*-test was used to determine statistical significance. In panels E and F, the intracellular c-di-GMP levels were determined using a cyclic-di-GMP assay kit (Lucerna, USA). Data are representative of two independent experiments with three technical replicates each and are shown as mean ± SD. ^*^P < 0.05, ^**^P < 0.01, ^***^P < 0.001, ^****^P < 0.0001; ns, not significant.

Given that temperature influences biofilm formation and that the functional amyloid Fap is the primary ECM component in *P. fluorescens* PF07, we hypothesized that *fap* gene expression is modulated by growth temperature. We quantified the transcriptional levels of each gene in the *fapA–F* gene cluster via quantitative reverse transcription-PCR (qRT-PCR) after 3 days of incubation at 28°C, 15°C, and 4°C and found that the transcription of all genes except *fapF* was significantly repressed by low temperatures (Fig. S2). Accordingly, *fapA* was selected as a representative of the transcriptional status of *fapA–E* for subsequent time-course experiments. As shown in Fig. 3A, *fapA* expression levels remained suppressed at 15°C and 4°C throughout the 13-day experimental period, relative to those at 28°C after 3 days of incubation. Despite a time-dependent increase in *fapA* expression with extended incubation, the *fapA* expression levels at 15°C and 4°C after 13 days of growth were only 24.60% and 16.05% of the levels measured at 28°C after 3 days, respectively. Flagellin FliC is associated with swimming motility (30), and alkaline protease AprA is the primary exoprotease produced by *Pseudomonas* (31, 32). In contrast to *fapA*, *fliC,* and *aprA* expression at 15°C and 4°C was significantly increased relative to 28°C after 3 days of incubation (Fig. 3B and C). Furthermore, we investigated the effect of a low-to-high temperature shift on the expression of these genes. PF07 colonies were cultured on tryptone plates at 15°C and 4°C for 3 days, then shifted to 28°C for an additional 1 day. As shown in Fig. 3D, *fapA* expression in temperature-shifted cultures increased 1.54-fold and 8.66-fold, respectively, relative to non-shifted control cultures. Conversely, the 28°C shift significantly reduced *fliC* and *aprA* expression. These findings indicate that low temperatures repress *fapA* expression while promoting *fliC* and *aprA* expression.

**Fig 4:**
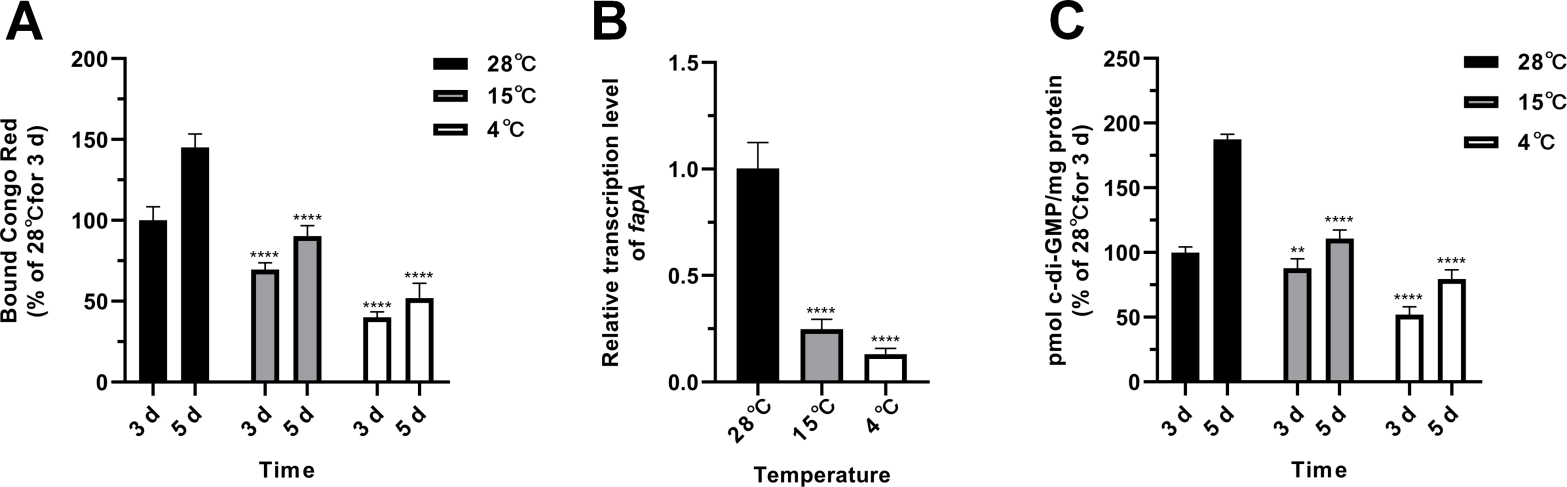
Low temperature represses ECM production, *fapA* transcription, and intracellular c-di-GMP content in macrocolony biofilms of *P. fluorescens* PF07 grown on sterile salmon muscle juice agar plates. (**A**) Quantification of ECM content in PF07 macrocolony biofilms cultured at different temperatures via the Congo red binding assay. ECM levels are percentages relative to those measured at 28°C for 3 days. Data are presented as mean ± SD of three biological replicates with three technical replicates each. Two-way ANOVA followed by Dunnett’s multiple comparisons test was used to determine statistical significance compared to the 28°C group at each time point. (**B**) qRT-PCR analysis of *fapA* transcription levels in 3-day-old macrocolony biofilms of PF07 cultured at different temperatures. The expression levels are relative to those at 28°C. Data are mean ± SD of three biological replicates with three technical replicates each. One-way ANOVA with Dunnett’s T3 multiple comparison test was used to determine statistical significance compared to the levels at 28°C. (**C**) Quantification of intracellular c-di-GMP levels in PF07 macrocolonies cultured at different temperatures. The c-di-GMP levels are expressed as a percentage relative to those at 28°C for 3 days. Data are mean ± SD of two biological replicates with three technical replicates each. Two-way ANOVA followed by Dunnett’s multiple comparisons test was used to determine statistical significance compared to the 28°C group at each time point. ^**^P < 0.01, ^****^P < 0.0001.

Our recent work demonstrated that *fapA* transcription is directly regulated by BrfA, an enhancer-binding protein that directly senses c-di-GMP in PF07 (20), a finding we further validated here. As shown in Fig. S3, *brfA* deletion at 28°C led to significant downregulation of *fapA* expression and abrogated normal macrocolony biofilm formation, whereas *brfA* hyperactivation at 15°C promoted *fapA* expression and rescued the formation of macrocolony biofilms. Based on the identified c-di-GMP/BrfA/*fapA* regulatory pathway and the repression of *fapA* transcription by low temperatures, we hypothesize that low temperatures reduce intracellular c-di-GMP levels in PF07. Intracellular c-di-GMP levels in PF07 were quantified over time at varying incubation temperatures on tryptone plates. The results revealed that c-di-GMP levels at 28°C were significantly higher than those at 15°C and 4°C (Fig. 3E), and a temperature shift from 4°C to 28°C significantly induced c-di-GMP production (Fig. 3F). To mimic the nutrient conditions of fish processing and preservation, PF07 was cultured on sterilized salmon muscle juice agar plates under these low temperatures. It was found that ECM synthesis, *fapA* transcription, and intracellular c-di-GMP production were also inhibited at low temperatures (Fig. 4).

Taken together, *fapA* expression and intracellular c-di-GMP levels exhibited a temperature-dependent pattern consistent with that of biofilm formation, indicating that low-temperature inhibition of Fap-dependent biofilm formation in PF07 is mediated by reduced intracellular c-di-GMP levels.

### The DGC DebA and the PDE BifA are crucial for modulating intracellular c-di-GMP levels, *fapA* expression, and biofilm formation

**Fig 5:**
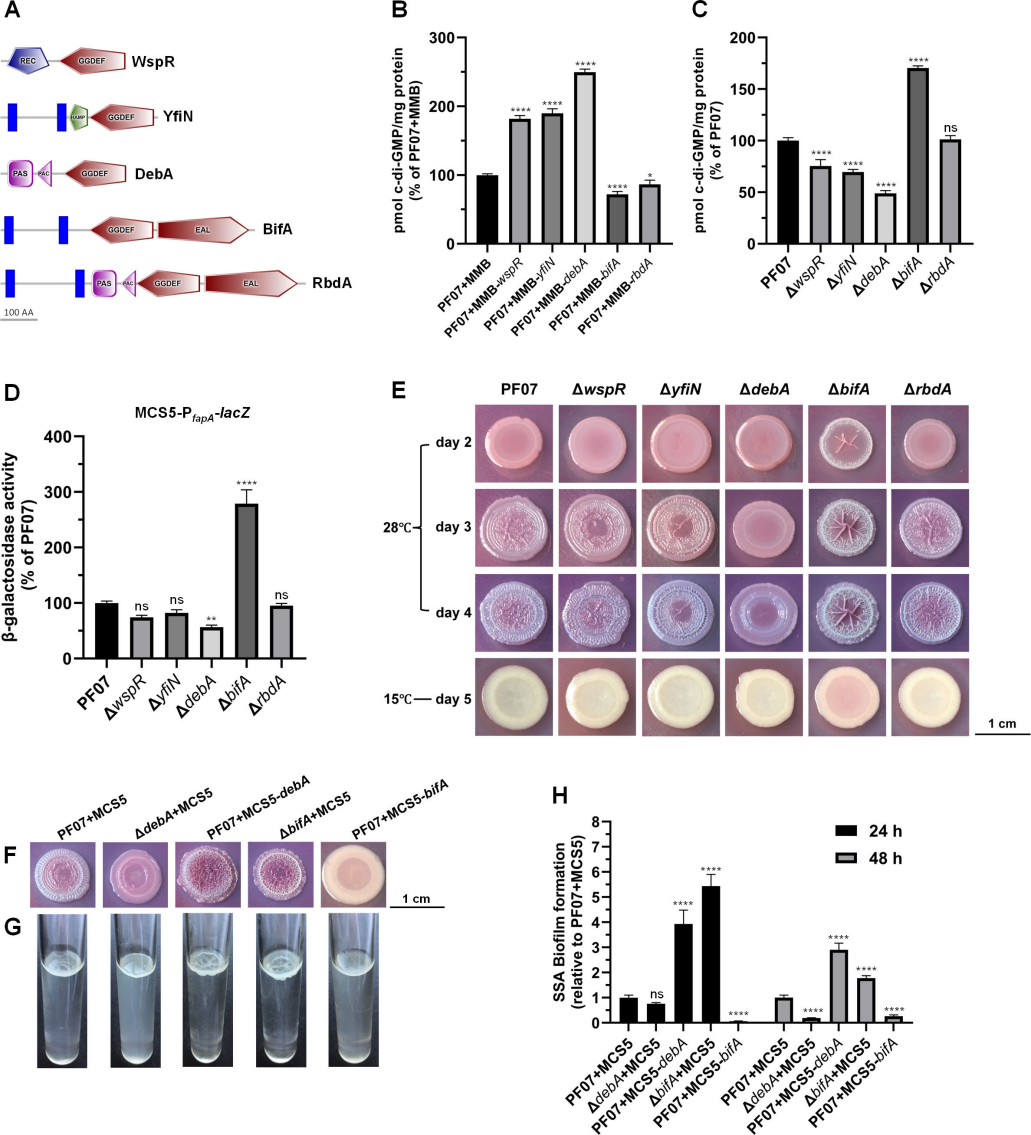
DebA and BifA are critical for regulating intracellular c-di-GMP levels, *fapA* expression, and biofilm formation in *P. fluorescens* PF07. (**A**) Domain architecture prediction of WspR, YfiA, DebA, BifA, and RbdA in PF07 (GenBank accession numbers XTS04226.1, XTS04227.1, XTS04228.1, XTS04229.1, and XTS04230.1) using the publicly available SMART algorithm (34). The REC (receiver) domain is conserved in response regulators; the HAMP domain (found in histidine kinases, adenylyl cyclases, methyl-binding proteins, and phosphatases) is a signal transduction domain; the GGDEF (diguanylate cyclase) domain is conserved in proteins that produce c-di-GMP from two molecules of GTP; the EAL domain is found in c-di-GMP-degrading phosphodiesterases; the PAS domain is a signal transduction module, and the PAC domain contributes to the PAS fold—these two domains are often combined and collectively termed the PAS domain (35). Predicted membrane-spanning helices are shown as blue vertical boxes. (**B**) Intracellular c-di-GMP levels in strains overexpressing *wspR*, *yfiA*, *debA*, *bifA*, or *rbdA*. The five genes were expressed from the IPTG-inducible vector pMMB206Gm in PF07. The strains were cultured at 28°C on tryptone agar plates with 0.5 mM IPTG for 2 days. (**C**) Intracellular c-di-GMP levels were quantified in *wspR*, *yfiA*, *debA*, *bifA*, or *rbdA* mutants cultured on tryptone agar plates at 28°C for 2 days. (**D**) *fapA* transcription levels measured using a β-galactosidase activity assay. The reporter plasmid MCS5-P*_fapA_-lacZ* was transferred to wild-type PF07 or the indicated mutant strains. Colonies formed after 2 days of growth at 28°C were used to measure β-galactosidase activity. c-di-GMP levels and β-galactosidase activity are percentages relative to those of the wild-type control (PF07+MMB or PF07). Data are representative of two independent experiments with three technical replicates each and are shown as mean ± SD. One-way ANOVA was used to determine statistical significance compared to the wild-type control, followed by Dunnett’s multiple comparison test (^*^P < 0.05, ^**^P < 0.01, ^****^P < 0.0001; ns, not significant). (**E**) Macrocolony biofilms of wild-type PF07 and Δ*wspR*, Δ*yfiN*, Δ*debA*, Δ*bifA*, and Δ*rbdA* mutants on Congo red plates (scale bar = 1 cm). Representative images from at least three biological replicates are shown. (**F**) Macrocolony biofilms of the indicated strains formed on Congo red plates after 4 days of growth at 28°C (scale bar = 1 cm). (**G**) Pellicles of indicated strains formed at the air-liquid interface of static cultures after 3 days of growth at 28°C. Macrocolony biofilm and pellicle images are representative of three biological replicates. (**H**) SSA biofilm formation of the indicated strains. Biofilms were formed by strains in 96-well microplates cultured at 28°C for 24 and 48 h with gentle shaking (150 rpm). The biofilms were quantified by crystal violet staining (OD_595_) and normalized to planktonic cell growth (OD_600_). The amount of biofilm is expressed as a percentage relative to that of PF07+MCS5. Data are mean ± SD of eight biological replicates. Two-way ANOVA with Dunnett’s multiple comparison test was used to determine statistical significance compared to PF07+MCS5 (^****^P < 0.0001; ns, not significant).

The critical DGCs and PDEs governing intracellular c-di-GMP concentrations and subsequent biofilm formation in PF07 have not been identified to date. In previous work, we identified a predicted DGC gene in PF07 with markedly upregulated expression during macrocolony biofilm development (19); we thus hypothesized its involvement in biofilm regulation and designated this gene *debA* in the present study. *Pseudomonads* typically harbor multiple DGC and PDE genes. WspR and YfiN are well-characterized DGCs that regulate biofilm formation in *Pseudomonads*, whereas BifA and RbdA are representative PDEs that mediate biofilm dispersal (13, 33). Accordingly, we selected PF07 homologs of these proteins for comparative analysis with DebA (Fig. 5A). DebA was predicted to contain PAS, PAC, and GGDEF domains. A BLAST search of the DebA amino acid sequence against the *Pseudomonas* Genome Database (https://www.pseudomonas.com/) was performed using the following criteria: ≥ 60% query coverage, ≥ 60% identity, and E-value ≤ 1.0E−12. The results revealed the presence of DebA-type proteins in 24.17% (320/1324) of the complete *Pseudomonas* genomes, including *P. fluorescens*, *P. extremaustralis*, and *P. putida*. To validate the *in vivo* catalytic functions of these DGCs and PDEs, the corresponding genes were overexpressed in PF07 using an isopropyl β-D-1-thiogalactopyranoside (IPTG)-inducible vector, pMMB206Gm, and intracellular c-di-GMP levels were subsequently quantified. As depicted in Fig. 5B, *wspR*, *yfiN*, and *debA* overexpression strains (PF07+MMB *wspR*, PF07+MMB *yfiN*, and PF07+MMB *debA*, respectively) exhibited significantly elevated c-di-GMP levels compared to the control strain (PF07+MMB) after induction with 0.5 mM IPTG. Notably, *debA* overexpression produced the highest c-di-GMP levels. Conversely, *bifA* and *rbdA* overexpression strains (PF07+MMB *bifA* and PF07+MMB *rbdA*, respectively) produced significantly lower c-di-GMP levels relative to the control strain, with *bifA* overexpression causing a greater reduction than *rbdA*. These findings demonstrate that WspR, YfiN, and DebA possess DGC activity, with DebA exhibiting the highest activity, and that BifA and RbdA possess PDE activity, with BifA demonstrating greater activity than RbdA.

We then sought to ascertain the significance of these DGCs and PDEs in modulating intracellular c-di-GMP levels and the subsequent regulation of *fapA* expression in PF07. In-frame deletion mutants of *wspR*, *yfiN*, *debA*, *bifA*, and *rbdA* were constructed (Fig. S4), and their c-di-GMP levels were quantified after 2 days of culture at 28°C. As illustrated in Fig. 5C, c-di-GMP levels were significantly reduced in all three DGC mutant strains (Δ*wspR*, Δ*yfiN*, and Δ*debA*) compared to the wild-type strain, with Δ*debA* exhibiting the lowest level, at 48.99% of the wild-type level. For the two PDE deletion mutants (Δ*bifA* and Δ*rbdA*), only Δ*bifA* exhibited significantly increased c-di-GMP levels relative to the wild-type strain, reaching 170.49%. Additionally, a *fapA* promoter-*lacZ* fusion reporter plasmid was used to assess *fapA* expression levels in the mutant strains (Fig. 5D). Deletion of *debA* reduced the *fapA* promoter activity to 56.66% of the wild-type strain, whereas deletion of *bifA* increased promoter activity to 278.71% of the wild-type strain. In contrast, deleting *wspR*, *yfiN*, or *rbdA* did not significantly affect *fapA* expression. These findings indicate that DebA and BifA are critical for modulating intracellular c-di-GMP levels and *fapA* expression at 28°C.

Given the established influence of intracellular c-di-GMP levels and *fap* expression on the macrocolony biofilm phenotype (20), biofilm morphology assays were conducted on Congo red plates with the mutant strains. As shown in Fig. 5E, the Δ*wspR*, Δ*yfiN*, and Δ*rbdA* mutants formed red and wrinkled macrocolonies after a 3-day incubation at 28°C and were phenotypically comparable to the wild-type PF07. However, Δ*debA* macrocolonies remained smooth on day 3 and began wrinkling on day 4, exhibiting delayed and diminished wrinkle formation, while Δ*bifA* macrocolonies exhibited wrinkling by day 2, with accelerated and enhanced wrinkling. In addition, all strains formed white colonies with a smooth morphology at 15°C, with the exception of Δ*bifA*, which developed a slightly reddish hue after 5 days of incubation. These observations demonstrate that DebA and BifA are essential DGC and PDE, respectively, for the formation of the red and wrinkled macrocolonies.

The influence of DebA and BifA on the formation of diverse biofilm types was also investigated. The full-length *debA* and *bifA* genes, each under the control of their native promoters, were overexpressed in the wild-type PF07 using the expression vector pBBR1MCS-5, generating PF07+MCS5 *debA* and PF07+MCS5 *bifA*, respectively. The empty vector was also introduced into the wild-type PF07, Δ*debA*, and Δ*bifA* strains, generating PF07+MCS5, Δ*debA*+MCS5, and Δ*bifA*+MCS5, respectively. Macrocolony biofilms, pellicles, and SSA biofilms of the five strains were comparatively analyzed at 28°C. Relative to the wild-type control (PF07+MCS5), PF07+MCS5 *debA* and Δ*bifA*+MCS5 colonies exhibited increased wrinkling after 4 days of growth, whereas Δ*debA*+MCS5 and PF07+MCS5 *bifA* colonies showed reduced wrinkling, particularly PF07+MCS5 *bifA*, which formed pale and smooth colonies (Fig. 5F). Similarly, PF07+MCS5 *debA* and Δ*bifA*+MCS5 produced thicker and more wrinkled pellicles compared to PF07+MCS5, whereas Δ*debA*+MCS5 and PF07+MCS5 *bifA* exhibited turbid growth with impaired pellicle formation (Fig. 5G). After 24 and 48 h of incubation in 96-well microplates, SSA biofilm biomass of PF07+MCS5 *debA* and Δ*bifA*+MCS5 increased to 1.78–5.43 times that of PF07+MCS5, while that of PF07+MCS5 *bifA* was reduced to 6.29% and 26.25% of PF07+MCS5, respectively. The biofilm biomass of Δ*debA*+MCS5 was comparable to that of PF07+MCS5 after 24 h but decreased to 18.71% of PF07+MCS5 after 48 h (Fig. 5H). These findings highlight the critical roles of DebA and BifA in macrocolony biofilm, pellicle, and SSA biofilm formation at 28°C.

### Low temperature primarily represses DebA protein expression and promotes certain PDE expression

**Fig 6:**
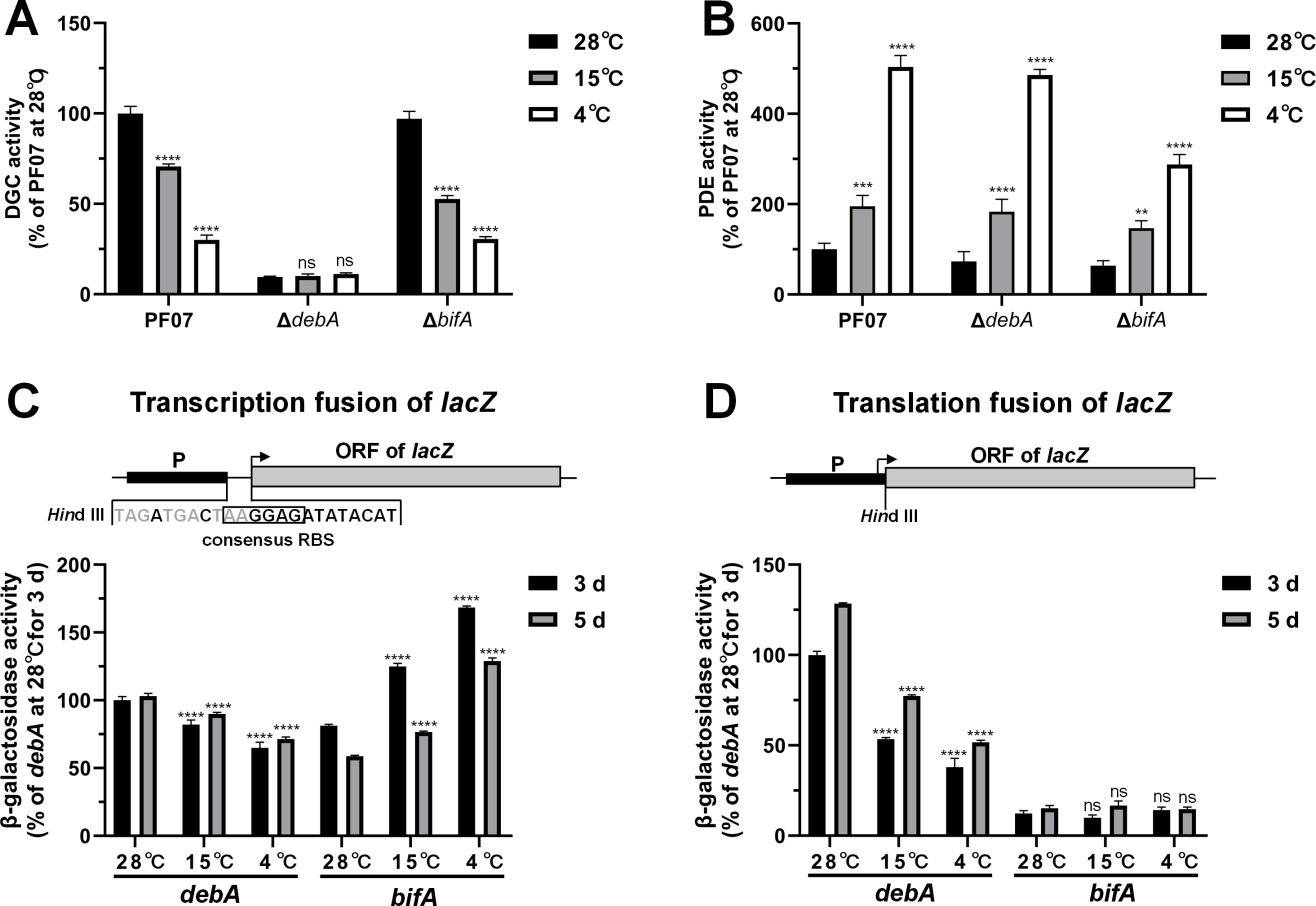
Expression analysis of *debA* and *bifA* genes in PF07 under different temperatures. (**A and B**) The Δ*debA*, Δ*bifA* mutants, and the wild-type PF07 were cultured at different temperatures for 3 days, followed by the determination of total diguanylate cyclase (DGC) (**A**) and phosphodiesterase (PDE) activities (**B**) at 28°C. The enzyme activities are presented as percentages relative to those of PF07 cultured at 28°C. Data are representative of at least two independent experiments with three technical replicates each and are shown as mean ± SD. Two-way ANOVA followed by Dunnett’s multiple comparison test was used to determine statistical significance compared to PF07 cultured at 28°C (^**^P < 0.01, ^***^P < 0.001, ^****^P < 0.0001; ns, not significant). (**C and D**) Transcriptional and translational levels of *debA* and *bifA* at different temperatures were detected using transcriptional fusion (**C**) and translational fusion (**D**) with *lacZ*. In transcriptional fusion vectors, a short sequence containing a *Hin*d III site, three tandem stop codons, and a consensus ribosomal binding site (RBS) was inserted upstream of the *lacZ* open reading frame (ORF), whereas only the *Hin*d III site was retained in translational fusion vectors. *debA* or *bifA*, including its native promoter and partial ORF, was inserted into the *Hin*d III site to generate transcriptional/translational fusion with *lacZ*. The arrows indicate translation initiation codon positions. The reporter vectors were introduced into wild-type PF07, with β-galactosidase activity assayed to evaluate the transcriptional or translational levels. Values are percentages relative to the transcriptional or translational levels of *debA* at 28°C for 3 days. Data are representative of at least two independent experiments with three technical replicates each and are shown as mean ± SD. Two-way ANOVA was used to determine statistical significance compared to the levels at 28°C for 3 days, followed by Dunnett’s multiple comparison test (^****^P < 0.0001; ns, not significant).

Given that DebA and BifA are crucial for modulating intracellular c-di-GMP levels, *fapA* expression, and biofilm formation in PF07, we hypothesized that temperature may control biofilm formation by regulating the expression of DebA and BifA. To investigate whether incubation temperature modulates intracellular DGC and PDE activities by regulating *debA* and *bifA* expression, wild-type PF07, Δ*debA*, and Δ*bifA* strains were cultured at 28°C, 15°C, and 4°C, and the total DGC/PDE activities of these strains were assayed at 28°C. For DGC activity (Fig. 6A), deletion of *debA* resulted in significantly lower total intracellular DGC activity than that of the wild-type strain across all three culture temperatures, with activity levels as low as 9.58% of the wild-type when cultured at 28°C. This result is consistent with the findings in Fig. 5, further confirming that DebA plays a critical role in regulating intracellular c-di-GMP synthesis. In addition, total DGC activities in wild-type PF07 and the Δ*bifA* mutant cultured at 15°C or 4°C were significantly lower than those in the 28°C controls, whereas Δ*debA* DGC activity was unresponsive to temperature fluctuations. These results suggest that low temperatures reduce intracellular DGC activity primarily by suppressing *debA* expression. For PDE activity (Fig. 6B), *bifA* deletion led to a marked decrease in total intracellular PDE activity regardless of culture temperature. This finding also aligns with the results in Fig. 5, further verifying that BifA is a key PDE mediating intracellular c-di-GMP degradation. Meanwhile, total intracellular PDE activity in wild-type PF07 and the Δ*debA* mutant cultured under the low temperature conditions was significantly higher than that in their 28°C counterparts, and deletion of *bifA* did not abrogate the temperature responsiveness of PDE activity. These results suggest that low-temperature cultivation enhances total intracellular PDE activity, likely via inducing the expression of additional PDE genes.

To further clarify how incubation temperature affects *debA* and *bifA* expression, we constructed transcriptional and translational fusion vectors in which each target gene—including its native promoter and partial open reading frame (ORF)—was fused to the *lacZ* reporter gene. PF07 strains harboring these reporter vectors were cultured at 28°C, 15°C, and 4°C, and β-galactosidase activity assays were performed to assess the effects of incubation temperature on *debA* and *bifA* expression at both the transcriptional (Fig. 6C) and translational levels (Fig. 6D). The results show that, compared with the 28°C controls, low temperatures (15°C and 4°C) significantly repressed *debA* transcription and translation. In contrast, low temperatures strongly promoted *bifA* transcription but had no significant effect on its translation. Additionally, our qRT-PCR results also confirm that low temperatures significantly repress the transcription of *debA* while promoting that of *bifA* (Fig. S5). Collectively, these data indicate that low temperatures markedly reduce DebA protein levels but do not affect BifA protein levels.

Taken together, our findings demonstrate that low temperatures reduce total intracellular DGC activity primarily by repressing DebA protein expression, while simultaneously enhancing total intracellular PDE activity via upregulating the expression of certain uncharacterized PDE genes.

### DebA harbors a PAS domain that enhances its thermal stability at elevated temperatures and exhibits a higher optimal temperature than BifA

**Fig 7:**
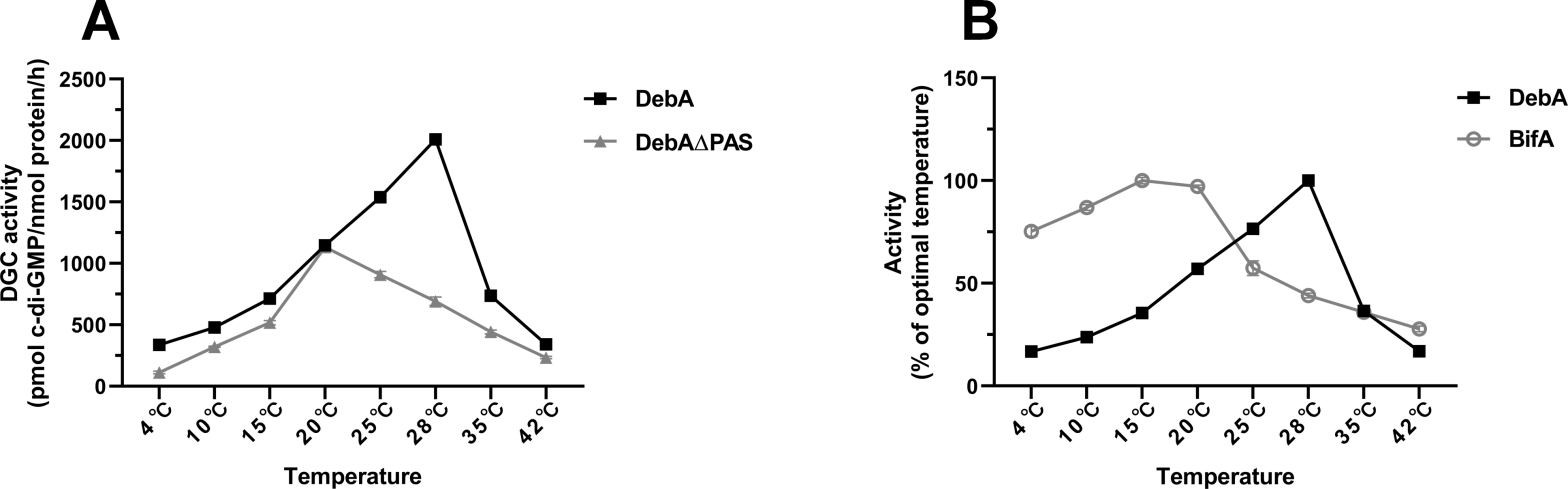
Temperature-dependent enzymatic activity of purified DebA, DebAΔPAS, and BifA proteins. (**A**) DGC activities of DebA and DebAΔPAS are expressed as c-di-GMP produced per nmol of purified protein per hour. (**B**) The enzymatic activities of DebA and BifA at different temperatures are presented as percentages of their activities measured at their respective optimal temperatures. Data are representative of at least two independent experiments with three technical replicates each and are shown as mean ± SD.

DebA harbors an N-terminal PAS domain, a structural module typically involved in environmental signal sensing to modulate enzymatic properties (2, 24). To characterize the role of this domain in DebA function, we heterologously expressed and purified N-terminally His-tagged full-length DebA and its PAS domain deletion mutant (DebAΔPAS) (Fig. S6), followed by performing temperature-dependent enzymatic activity assays over a temperature range of 4°C–42°C (Fig. 7A). Both enzymes exhibited a characteristic bell-shaped activity profile, with activity increasing to an optimal temperature before declining due to thermal denaturation. However, full-length DebA reached a maximum activity at 28°C, whereas DebAΔPAS peaked at 20°C, representing an 8°C reduction in the optimal temperature. Notably, DebA and DebAΔPAS displayed comparable DGC activity at 20°C; DebAΔPAS activity declined immediately above this temperature, while DebA retained elevated activity up to 28°C, at which point it exhibited a maximal activity advantage relative to DebAΔPAS. These results demonstrate that the PAS domain can enhance DebA’s thermal stability and DGC activity at elevated temperatures.

To further compare the thermal tolerance of DebA and BifA, we also purified a truncated BifA lacking its predicted transmembrane region (Fig. S6) and conducted parallel temperature-dependent activity assays with the purified DebA and BifA proteins (Fig. 7B). The data revealed distinct thermal preferences between the two enzymes: DebA displayed an optimal reaction temperature of 28°C, whereas BifA exhibited a substantially lower optimal temperature of 15°C. At 28°C, DebA maintained peak catalytic activity, while BifA activity was reduced to 44.03% of its maximum level. Conversely, at 4°C, DebA retained merely 16.79% of its peak activity, whereas BifA maintained 75.26% of its maximal activity at this low temperature. These results indicate that BifA exhibits typical characteristics of a cold-adapted enzyme, including high catalytic activity at low temperatures and poor thermostability at elevated temperatures (36). In contrast, DebA displays low activity at low temperatures and high thermostability at 28°C. This striking discrepancy in thermal tolerance between DebA and BifA can amplify the effects of temperature fluctuations on intracellular c-di-GMP pools.

## DISCUSSION

This investigation sought to elucidate the influence of temperature on biofilm development in *P. fluorescens* PF07, a psychrotrophic food spoiler known to produce Fap as a primary constituent of its biofilm matrix (19). Our findings indicated that reduced temperatures impede Fap-dependent biofilm formation by downregulating intracellular c-di-GMP levels. Furthermore, we identified a novel DGC (DebA) and a cold-adapted PDE (BifA), which are coordinately regulated by temperature at both the protein expression and enzymatic activity levels and, in turn, modulate biofilm formation in this strain.

Temperature is a critical environmental signal governing biofilm formation across numerous bacterial species. Our data demonstrate that reduced temperatures (15°C and 4°C) significantly attenuate macrocolony biofilm development and matrix production in *P. fluorescens* PF07 compared to 28°C (Fig. 1), a phenotype consistent with that of UK4, another Fap-producing *P. fluorescens* strain (Fig. S1). Low temperatures also suppress pellicle and SSA biofilm formation in PF07 (Fig. 2). These observations align with reports of temperature-dependent biofilm regulation in foodborne pathogens. For example, the impaired biofilm formation at low temperatures has been documented in *Listeria monocytogenes*, *Salmonella enterica* serotype Kentucky, and *Vibrio parahaemolyticus* (6, 29, 37). Notably, a previous study on *P. fluorescens* PF07 reported SSA biofilm phenotypes at low temperatures that contradict those observed in the present study (38). This discrepancy is most likely attributable to differences in experimental conditions: the authors cultured PF07 statically in a very small volume for an extended incubation period (200 μL, 196 h) and did not normalize biofilm biomass to planktonic cell density. In contrast, low temperatures promote biofilm formation in *V. cholerae* and *P. aeruginosa* (14, 39), highlighting interspecies variability in temperature-mediated biofilm regulation likely driven by divergent adaptive strategies to temperature fluctuations.

The mechanism by which low temperature inhibits biofilm formation in PF07 was further investigated. We first found that *fap* gene expression and intracellular c-di-GMP levels were significantly decreased at low temperatures (15°C and 4°C) (Fig. 3 and 4). Our previous work demonstrated that PF07 employs the c-di-GMP/BrfA/Fap signaling pathway to regulate biofilm formation (20), a finding that is further validated in the present study (Fig. S3). These results indicate that low temperature inhibits *fap* gene expression and biofilm formation via the c-di-GMP signaling pathway. However, temperature exerts an opposite regulatory pattern on the expression of *fliC* and *aprA* compared with that of *fapA* (Fig. 3A through D). The *fliC* gene is regulated by the transcriptional regulator FleQ, whose activity has been shown to be inhibited by c-di-GMP in *P. aeruginosa* (30, 40). Temperature may therefore also control *fliC* expression and bacterial motility in PF07 by modulating intracellular c-di-GMP levels and FleQ activity. The production of AprA was reported to be significantly reduced when Fap was overexpressed in *P. aeruginosa* (32); thus, the opposite expression patterns of *fapA* and *aprA* in PF07 are expected. How temperature regulates *aprA* expression remains to be elucidated in future work.

Intracellular c-di-GMP levels in bacteria are typically modulated by multiple DGCs and PDEs. In PF07, we found that DebA and BifA play more critical roles in regulating intracellular c-di-GMP levels, *fap* gene expression, and biofilm formation compared with the well-characterized c-di-GMP metabolic enzymes WspR, YfiN, and RbdA (Fig. 5). The results shown in Fig. 5B confirmed that DebA and BifA exhibit enhanced enzymatic activity, which is likely the primary driver of this observed functional discrepancy. Distinct c-di-GMP metabolic enzymes may also exhibit divergent regulatory functions due to differences in their environmental signal responsiveness, subcellular localization, and protein–protein interactions (2, 12, 41). We further found that temperature modulates intracellular c-di-GMP levels by coordinately regulating the abundance of DGCs and PDEs. Specifically, low temperatures reduce intracellular DGC activity primarily by suppressing *debA* expression (Fig. 6A, C, and D). In contrast, low temperatures robustly upregulate *bifA* transcription but exert no significant effect on its translation, instead modulating the expression of certain other uncharacterized PDE genes (Fig. 6B through D). In *Escherichia coli*, temperature regulates the expression of several DGC and PDE genes by controlling the level of RpoS, a global regulator of stationary-phase responses (42). In *P. putida*, *bifA* transcription is subject to cascading regulation by two transcription factors, FleQ and FliA (27, 30). In PF07, the transcription of *bifA* is also likely regulated by these two factors. However, the discordance exists between the transcription and translation of *bifA*, suggesting the involvement of post-transcriptional or translational-level regulation in this process. The specific molecular mechanisms by which temperature modulates the expression of *debA* and *bifA* in PF07 deserve further investigation in future studies.

Finally, we revealed that temperature regulates intracellular c-di-GMP levels through differential modulation of DebA and BifA enzymatic activities in PF07, a regulatory mode that has not been previously reported. DebA harbors a PAS domain that enhances its thermal stability at elevated temperatures and endows DebA with a higher optimal temperature than BifA (Fig. 7). This function of the PAS domain shares similarities with the thermosensory PAS domain identified in the DGC TdcA from *P. aeruginosa* (24), as both domains contribute to increased enzyme activity at elevated temperatures. However, DebA and TdcA exhibit distinct rate-temperature dependencies, as quantified by the *Q*_10_ temperature coefficient (the fold change in reaction rate with a 10°C increase in temperature). In the present study, DebA exhibits a *Q*₁₀ value of ~2, consistent with the typical rate-temperature dependencies of most enzymes (24). In contrast, the PAS domain of TdcA confers an extremely high *Q*₁₀ value of 135, a value analogous to that of thermosensitive transient receptor potential proteins, thus validating its designation as a thermosensory PAS domain. By comparison, the enhancement of enzymatic activity at elevated temperatures by the PAS domain of DebA is less prominent than that mediated by the thermosensitive PAS domain of TdcA. Furthermore, we report the first identification of BifA as a canonical cold-adapted PDE from the psychrotrophic strain PF07. Most cold-adapted enzymes retain high catalytic activity at 20°C–25°C and maintain over 40%–50% of their maximum activity at 0–10°C (43). BifA has an optimal temperature of 15°C and retains 75.25% of its maximum activity at 4°C, demonstrating superior cold tolerance compared to most characterized cold-adapted enzymes. Psychrophilic and psychrotrophic microorganisms typically adapt to low-temperature environments by evolving cold-adapted enzymes, which compensate for the adverse effects of low temperatures by increasing structural flexibility. This structural flexibility may be associated with the entire protein or specific structural regions, particularly those involved in catalysis (36, 44). Taken together, the PAS domain of DebA enhances its thermal stability, and BifA exhibits exceptional cold tolerance; these properties together enable temperature to differentially regulate their enzymatic activities. This coordinated regulation ultimately achieves the precise control of intracellular c-di-GMP levels in PF07.

**Fig 8:**
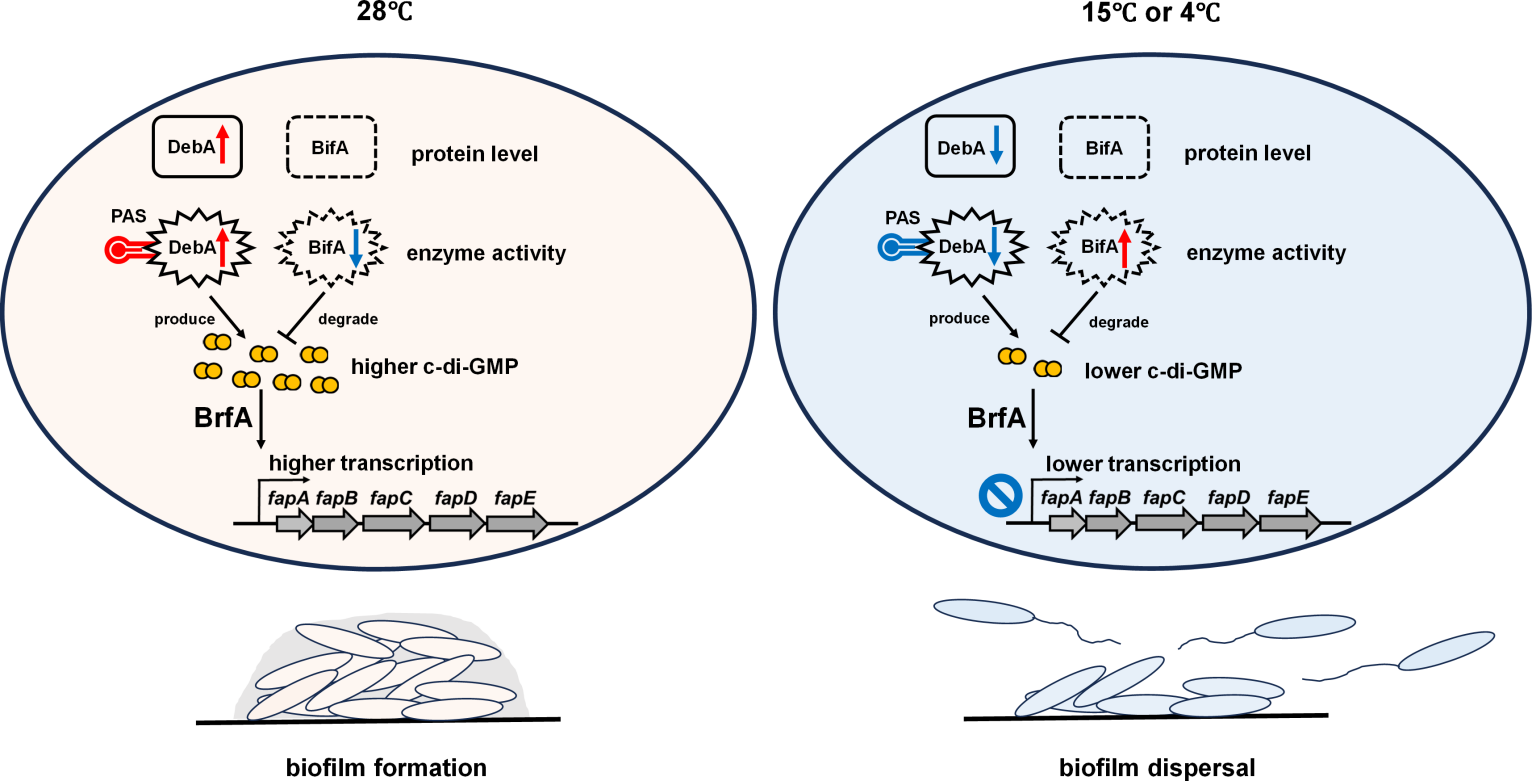
A temperature-dependent regulatory model for c-di-GMP-mediated biofilm formation in *P. fluorescens* PF07. At 28°C, high DebA expression and PAS domain-dependent catalytic activity, paired with thermolabile BifA, elevate intracellular c-di-GMP levels, which promote robust biofilm formation via the c-di-GMP/BrfA/Fap pathway. At 15°C or 4°C, reduced DebA expression and activity, alongside cold-stable BifA, decrease c-di-GMP synthesis and accelerate its degradation, leading to low intracellular c-di-GMP levels that repress *fap* expression and inhibit biofilm formation.

Integrating the data from the present study, we propose a novel regulatory model in which temperature coordinately regulates DebA and BifA at both the protein expression and enzymatic activity levels to modulate intracellular c-di-GMP levels and subsequent biofilm formation (Fig. 8). At a relatively high temperature (28°C), DebA is highly expressed and maintains robust catalytic activity via its PAS domain, whereas BifA exhibits low activity due to its thermolability. This combinatorial effect enhances c-di-GMP synthesis and reduces its degradation, leading to elevated intracellular c-di-GMP levels that promote biofilm formation via the c-di-GMP/BrfA/Fap pathway. In contrast, at low temperatures (15°C and 4°C), DebA expression and activity are diminished, while BifA retains high activity owing to its high cold tolerance. This functional shift decreases c-di-GMP synthesis and accelerates its degradation, yielding low intracellular c-di-GMP levels that consequently inhibit *fap* expression and biofilm formation. This study reveals a novel model by which temperature governs biofilm formation via the c-di-GMP signaling pathway in the psychrotrophic food spoiler *P. fluorescens* PF07. This regulatory process centers on the coordinated regulation of the expression and enzymatic activity of two key enzymes: the novel DGC DebA and the cold-adapted PDE BifA, orthologs of which are widely distributed across *Pseudomonas* species. These findings thus identify promising molecular targets for the development of strategies to combat biofilm formation by psychrotrophic pseudomonads in food ecosystems.

## MATERIALS AND METHODS

### Bacterial strains and growth conditions

The *E. coli* strains, *P. fluorescens* strains, and plasmids used in this study are detailed in Table 1. *P. fluorescens* strains were generally grown in Luria-Bertani broth (LB) or tryptone broth (10 g/L tryptone) at 28°C. *E. coli* S17-1/*λpir* was used for cloning and conjugation and was routinely cultured in LB medium at 37°C. *P. fluorescens* PF07 was cultured on sterile salmon muscle juice agar in the designated experiments. The preparation of this sterile agar was carried out according to the method described by Dalgaard (45) with minor modifications. Briefly, salmon pieces were homogenized with 100 mL of sterile water per 100 g of tissue. The homogenate was boiled for 5 min, filtered through gauze, and centrifuged at 4,696 × *g* for 30 min. To the resulting supernatant, 0.1 M phosphate buffer (pH 6.8) and 1.5% (wt/vol) agar were added; the mixture was then sterilized at 105°C for 10 min to yield sterile salmon muscle juice agar. To compensate for dilution and heat decomposition, L-cysteine and L-methionine were supplemented to a final concentration of 40 mg/L each. Antibiotics were used as needed at the following activity-corrected concentrations: for *E. coli*, gentamicin (Gm) at 10 μg/mL, tetracycline (Tc) at 10 μg/mL, and ampicillin (Ap) at 100 μg/mL; for *P. fluorescens*, Gm at 50 μg/mL for transconjugant screening, Gm at 5 or 10 µg/mL for plasmid maintenance, and Tc at 12 μg/mL for screening.

**TABLE 1:** Bacterial strains and plasmids used in this study

Strain or plasmid	Description	Reference or source
*Escherichia coli* strains		
BL21(DE3)	F^−^ *ompT hsd*S_B_ (r_B_^−^ m_B_^−^) *gal dcm* (DE3)	Lab stock
S17-1/λpir	*recA1 endA1 thiE1 pro-82 creC510 hsdR17* RP4-2(Km:Tn7 Tc:Mu-1), λpir, Tp^r^Sm^r^	(46)
*Pseudomonas fluorescens* strains		
PF07	An isolate from refrigerated large yellow croaker	(20)
UK4	Isolated from drinking water reservoir	DSMZ
Δ*debA*	PF07, *debA* deletion mutant	This work
Δ*wspR*	PF07, *wspR* deletion mutant	This work
Δ*yfiN*	PF07, *yfiN* deletion mutant	This work
Δ*bifA*	PF07, *bifA* deletion mutant	This work
Δ*rbdA*	PF07, *rbdA* deletion mutant	This work
Plasmids		
pT18mobsacB	Mobilizable vector for gene disruption, Tet^r^	Addgene
pT18-*debA*-updown	Suicide plasmid pT18mobsacB containing up and down homologous region of *debA*, Tet^r^	This work
pT18-*wspR*-updown	Suicide plasmid pT18mobsacB containing up and down homologous region of *wspR*, Tet^r^	This work
pT18-*yfiN*-updown	Suicide plasmid pT18mobsacB containing up and down homologous region of *yfiN*, Tet^r^	This work
pT18-*bifA*-updown	Suicide plasmid pT18mobsacB containing up and down homologous region of *bifA*, Tet^r^	This work
pT18-*rbdA*-updown	Suicide plasmid pT18mobsacB containing up and down homologous region of *rbdA*, Tet^r^	This work
pMMB206	Expression vector, IncQ *lacI*^q^ P*_tac-lac_ lacZα* Δ*bla cat* (Cm^r^)	(47)
pBBR1MCS-5	Expression vector, P*_lac_ lacZα*, Gm^r^	(48)
pMMB206Gm	pMMB206 Δ*cat gen* (Gm^r^)	This work
pMMB-*debA*	pMMB206Gm with *debA*, Gm^r^	This work
pMMB-*wspR*	pMMB206Gm with *wspR*, Gm^r^	This work
pMMB-*yfiN*	pMMB206Gm with *yfiN*, Gm^r^	This work
pMMB-*bifA*	pMMB206Gm with *bifA*, Gm^r^	This work
pMMB-*rbdA*	pMMB206Gm with *rbdA*, Gm^r^	This work
pMCS5-*debA*	pBBR1MCS-5 containing *debA* with its own promoter, Gm^r^	This work
pMCS5-*bifA*	pBBR1MCS-5 containing *bifA* with its own promoter, Gm^r^	(20)
pMCS5-*lacZ*	*lacZ* transcriptional fusion reporter vector derived from pBBR1MCS-5, Gm^r^	(20)
pMCS5::*lacZ*	*lacZ* translational fusion reporter vector derived from pBBR1MCS-5, Gm^r^	This work
pMCS5-P*_fapA_-lacZ*	*fapA* transcriptional fusion reporter vector derived from pMCS5-*lacZ*, Gm^r^	(20)
pMCS5-P*_debA_-lacZ*	*debA* transcriptional fusion reporter vector derived from pMCS5-*lacZ*, Gm^r^	This work
pMCS5-P*_bifA_-lacZ*	*bifA* transcriptional fusion reporter vector derived from pMCS5-*lacZ*, Gm^r^	This work
pMCS5-P*_debA_*::*lacZ*	*debA* translational fusion reporter vector derived from pMCS5-*lacZ*, Gm^r^	This work
pMCS5-P*_bifA_*::*lacZ*	*bifA* translational fusion reporter vector derived from pMCS5-*lacZ*, Gm^r^	This work
pET28a	T7/his-tag expression vector, Km^r^	Lab stock
pET28a::*debA*	pET28a harboring *debA* (bp 1-1014 of its ORF), Km^r^	This work
pET28a::*debA*ΔREC	pET28a harboring *debA*ΔPAS (bp 415-1014 of the *debA* ORF), Km^r^	This work
pET28a::*bifA*	pET28a harboring *bifA* (bp 544-2052 of its ORF)	This work

### Macrocolony biofilm observation

A 5-μL aliquot of an overnight LB culture was spotted onto Congo red agar plates (1% tryptone, 1.2% agar, 30 μg/mL Congo red, and 10 μg/mL Coomassie brilliant blue G250). The plates were incubated at 4°C, 15°C, 23°C, 28°C, 33°C, and 37°C for 13 days in high-precision temperature-controlled incubators (temperature fluctuation: ± 0.5°C). At least six biological replicates were performed for each strain, and macrocolony images were captured daily. In addition, macrocolonies incubated for 3, 5, and 13 days at the aforementioned temperatures on tryptone agar plates (without Congo red and Coomassie brilliant blue G250) were gently scraped from the plates and examined using TEM (Hitachi H-600, Japan) according to a previously described protocol (19).

### Matrix quantification

Matrix quantification was performed using the Congo red binding assay, a method described previously (2). Briefly, a 5-μL aliquot of an overnight preculture was spotted onto tryptone agar plates or fish juice agar plates and incubated at 28°C, 15°C, or 4°C for 3, 5, 9, and 13 days. Three colonies were scraped from the plates, resuspended in 1 mL phosphate-buffered saline (PBS) supplemented with 40 μg/mL Congo red dye, and incubated at 28°C for 1 h. Three parallel samples were prepared under each condition. Samples were centrifuged at 13,800 × *g* for 2 min, and the resulting supernatants were transferred to a clear 96-well plate. Absorbance at 490 nm was measured in triplicate using a plate reader (Tecan, Switzerland), with PBS containing 40 μg/mL Congo red serving as the no-matrix standard.

### Pellicle formation assay

Overnight cultures were inoculated at a 1:1,000 dilution into 6 mL of fresh sterile tryptone broth (~10^6^ cfu/mL). The cultures were incubated statically in glass tubes at 28°C, 15°C, or 4°C for 13 days. Photographs were captured daily to monitor pellicle formation at the air-liquid interface of the static cultures. Assays were performed in triplicate.

### Growth determination and crystal violet assay

An overnight culture was diluted 1:1,000 to a final concentration of ~10⁶ cfu/mL in tryptone broth. Subsequently, 2 mL of the dilution culture was added to a 24-well polystyrene microplate and incubated at 28°C, 15°C, or 4°C with shaking at 130 rpm. The optical density at 600 nm (OD_600_) was measured to determine bacterial growth at the following time points: 12, 24, 48, 72, 96, 120, 144, 168, 192, and 216 h. Biofilm formation in the 24-well microplates was quantified by crystal violet staining following a protocol similar to that used for 96-well microplates, as previously described (49). After the culture was removed, the wells were washed with running distilled water and stained with 2.5 mL of a 1% crystal violet solution for 15 min. The stained biofilms were washed thoroughly with running water and allowed to air dry. Crystal violet bound to the biofilms was dissolved in 2.5 mL of ethanol for 30 min, and biofilm formation was quantified by measuring the OD_595_. Eight biological replicates were used for each temperature condition at each time point, and the experiment was repeated at least twice independently.

### Extraction and quantification of intracellular c-di-GMP

c-di-GMP was extracted and quantified as previously described (20). Four macrocolonies cultured on tryptone agar plates or fish juice agar plates were scraped into 5 mL of PBS and washed once with PBS. The resulting pellets were resuspended in PBS and heated at 100°C for 5 min. Ethanol was then added to a final concentration of 65%. The samples were centrifuged at 4,696 × *g* for 10 min, and the supernatant was evaporated to dryness via vacuum freeze-drying. The dried samples were dissolved in RNase-free water to analyze the c-di-GMP content using a c-di-GMP assay kit (Lucerna, USA). The protein content of each pellet was determined using a Bradford protein assay kit (Beyotime, China) after resuspending the pellets in 5 mL of 0.1 M NaOH and heating at 95°C for 15 min. Intracellular c-di-GMP levels were normalized to the total protein content of each sample.

### RNA extraction, cDNA synthesis, and qRT-PCR

Total RNA was extracted from macrocolonies on tryptone plates using a TRIzol Plus RNA purification kit (Thermo Fisher, USA) according to the manufacturer’s instructions. Contaminating genomic DNA in the total RNA was removed using an RNase-free DNase set (Qiagen, Germany). cDNA was synthesized using SuperScript III First-Strand Synthesis SuperMix (Thermo Fisher, USA), and the resulting cDNA samples were subsequently analyzed by qRT-PCR using Power SYBR Green PCR master mix (Applied Biosystems, USA). The qRT-PCR primers are detailed in Table S1. The relative expression level of each target gene was calculated as 2^-ΔΔ^*^Ct^* (50), with the 16S rRNA gene serving as an internal control for normalization. All experiments were performed in triplicate using three independent biological cultures.

### Plasmid and strain construction

The broad-host-range expression vector pMMB206 was modified to construct pMMB206Gm (47), in which a gentamicin resistance gene replaced the chloramphenicol resistance gene. This was achieved by amplifying the gentamicin resistance gene from pBBR1MCS-5 via PCR using the Gm-3/4 primer pair and ligating the resulting gentamicin resistance fragment to *Dra*I-digested pMMB206 using a ClonExpress II one-step cloning kit (Vazyme, China). The expression vector pMMB206Gm was used to express *wspR*, *yfiA, debA*, *bifA*, and *rbdA* under the control of the IPTG-inducible promoter P*_taclac_*. The genes were PCR-amplified using the primer pairs *wspR*-A/B, *yfiN*-A/B, *debA*-A/B, *bifA*-A/B, and *rbdA*-A/B, and the resulting fragments were ligated to *Eco*RI-digested pMMB206Gm to yield pMMB-*wspR*, pMMB-*yfiN*, pMMB-*debA*, pMMB-*bifA*, and pMMB-*rbdA*.

Deletion mutants were constructed by allelic replacement with the suicide vector pT18mobsacB, as previously described (20). The expression plasmid pMCS5-*debA* was constructed to express the *debA* gene under the control of its native promoter. The complete *debA* gene with its native promoter was amplified using the primers *debA*-MCS-A/B and inserted into the *Hin*d III site of pBBR1MCS-5 (48).

The *lacZ* transcriptional fusion reporter vector pMCS5-*lacZ* was constructed in our previous work (20). The ORF of *lacZ* was preceded by no promoter sequence but fused to a short DNA fragment containing a single *Hin*d III restriction site, three tandem stop codons, and a consensus ribosomal binding site (RBS) (AAGCTTAGATGACTAAGGAGATATACAT). The translational fusion reporter vector pMCS5::*lacZ* was constructed by inverse PCR amplification of pMCS5-*lacZ* with the primer pair pHM25-1/2, followed by *Hin*d III digestion and self-ligation of the resulting amplicon; this vector lacked the aforementioned short fragment but retained the *Hin*d III site. The transcriptional and translational fusion fragments of *debA*, comprising its upstream regulatory and partial coding sequences, were PCR-amplified with the primer pairs *debA*-1/Z*debA*-2 and *debA*-1/F*debA*-2, respectively; those of *bifA* were amplified using the primer pairs *bifA*-1/Z*bifA*-2 and *bifA*-1/F*bifA*-2, respectively. Insertion of the transcriptional fusion fragments of both genes individually into the *Hind* III site of pMCS5-*lacZ* generated the transcriptional fusion reporter vectors pMCS5-P*_debA_-lacZ* and pMCS5-P*_bifA_-lacZ*. Separately cloning the translational fusion fragments into pMCS5::*lacZ* yielded the translational fusion reporter vectors pMCS5-P*_debA_::lacZ* and pMCS5-P*_bifA_::lacZ*.

Plasmids pET28a::*debA*, pET28a::*debA*ΔREC, and pET28a::*bifA* were constructed for heterologous expression of His-tagged DebA, DebAΔREC, and BifA proteins in *E. coli* BL21(DE3). PCR amplicons of *debA*, *debA*ΔREC, and *bifA* were generated using the primer pairs *debA*e-1/3, *debA*e-2/3, and *bifA*e-1/2, respectively, and subsequently cloned into the *Nde* I and *Xho* I restriction sites of pET28a.

All primers used in this study are listed in Table S1. All cloning steps involving PCR were verified by sequencing (Tsingke, Hangzhou, China). Plasmids were transferred to *P. fluorescens* strains by biparental mating using *E. coli* S17-1/λpir as the donor strain.

### Expression and purification of recombinant proteins

The N-terminal His-tagged proteins His-DebA (amino acid residues 1–337; full-length DebA), His-DebAΔPAS (amino acid residues 139–337; PAS domain and its auxiliary PAC domain truncated), and His-BifA (amino acid residues 182–683; predicted transmembrane region truncated) were heterologously expressed in *E. coli* BL21(DE3) harboring plasmids pET28a::*debA*, pET28a::*debA*ΔPAS, and pET28a::*bifA*, respectively. Overnight *E. coli* cultures were diluted 1:100 into 500 mL LB broth and shaken at 37°C to mid-log phase, after which recombinant protein expression was induced with 1 mM IPTG and incubated at 20°C for 12–14 h. Harvested cells were resuspended in lysis buffer (500 mM NaCl, 20 mM Tris-HCl, pH 8.0) and sonicated on ice. The soluble fraction was subsequently isolated by centrifugation at 4,696 × *g* and 4°C for 30 min, then filtered through a 0.45-μm pore-size filter. Target proteins were purified via Ni-NTA resin (Genscript, China), then concentrated and desalted with an Amicon Ultra-4 centrifugal filter unit (Merck, Germany). Protein concentrations were quantified with a Bradford Protein Assay Kit (Beyotime, China).

### *In vitro* DGC/PDE activity assay

DGC activity was determined as c-di-GMP produced per mg of total protein or per nmol of purified protein per hour, whereas PDE activity was assessed as c-di-GMP degraded using the same units, according to a previously described method with minor modifications (51). For the assay of total intracellular DGC/PDE activity, macrocolonies grown on tryptone plates at different temperatures for 3 days were scraped off, and soluble total protein was extracted via sonication for subsequent activity determination. DGC assays were performed in 100 μL reaction mixtures containing ~0.1 mg of protein and DGC buffer (50 mM Tris-HCl, pH 8.0, 50 mM NaCl, 20 mM MgCl₂). Reactions were initiated by adding 100 μM GTP substrate, with substrate-free samples included as controls. After incubation at 28°C for 2 h, reactions were terminated by heating at 100°C for 5 min. Mixtures were centrifuged at 16,200 × *g* for 15 min, and the supernatants were collected. The amount of c-di-GMP produced in the supernatants was quantified using a c-di-GMP detection kit (Lucerna, USA). For PDE activity assays, 100 μL reaction mixtures were supplemented with 5 mM MnCl₂ in addition to the DGC reaction system. Heat-inactivated samples (100°C for 5 min) served as controls, and reactions were initiated by adding 50 μM c-di-GMP substrate. Following incubation at 28°C for 2 h, reactions were terminated by heating at 100°C for 5 min, and the mixtures were centrifuged at 16,200 × *g* for 15 min. The supernatants were collected, and the reduction in c-di-GMP levels was determined using the same c-di-GMP detection kit. For temperature-gradient activity assays of purified DebA, DebAΔPAS, and BifA proteins (4°C–42°C), the reaction system was identical to that used for total enzyme activity assays. The final concentrations of DebA and DebAΔPAS were adjusted to ~0.5 µM, and that of BifA to ~0.02 µM. Reaction mixtures were preincubated at the designated temperatures for 5 min before reactions were initiated by adding the GTP substrate or the c-di-GMP substrate.

### β-Galactosidase assays

β-Galactosidase (LacZ) activity was quantified using a β-galactosidase activity assay kit (Sangon Biotech, China) as previously described (20). Briefly, 2-day-old macrocolonies of PF07 strains harboring a *lacZ* reporter plasmid cultured on tryptone plates were gently scraped into 1 mL of 0.9% NaCl. The samples were centrifuged and resuspended in 1 mL of extraction solution. The cells were disrupted by sonicating on ice, followed by centrifugation. The resulting supernatant was used to determine β-galactosidase activity. Experiments were performed in at least two independent biological replicates, with three technical replicates per sample.

### Statistical analysis

All statistical analyses were performed using GraphPad Prism software (version 8.0.2). When mean values of more than two treatments were compared, analysis of variance (ANOVA) with a suitable *post hoc* test was performed. When the mean values of only two treatments were compared, an unpaired *t*-test was used. Statistical significance was defined as *P* < 0.05.

## SUPPLEMENTAL MATERIAL

Supplemental material aem.02387-25-s0001.docx Fig. S1 to S5; Table S1.

ASM does not own the copyrights to Supplemental Material that may be linked to, or accessed through, an article. The authors have granted ASM a non-exclusive, world-wide license to publish the Supplemental Material files. Please contact the corresponding author directly for reuse.
