# Supplementary material for: Thermoregulation of functional amyloid Fap-dependent biofilm formation via cyclic diguanosine monophosphate signaling in Pseudomonas fluorescens
Source: Appl Environ Microbiol. 2026 Mar 20;92(4):e02387-25. doi: 10.1128/aem.02387-25 (PMC13101488; doi:10.1128/aem.02387-25)
Supplement: Supplemental material — Fig. S1 to S5; Table S1. [file aem.02387-25-s0001.docx]

**
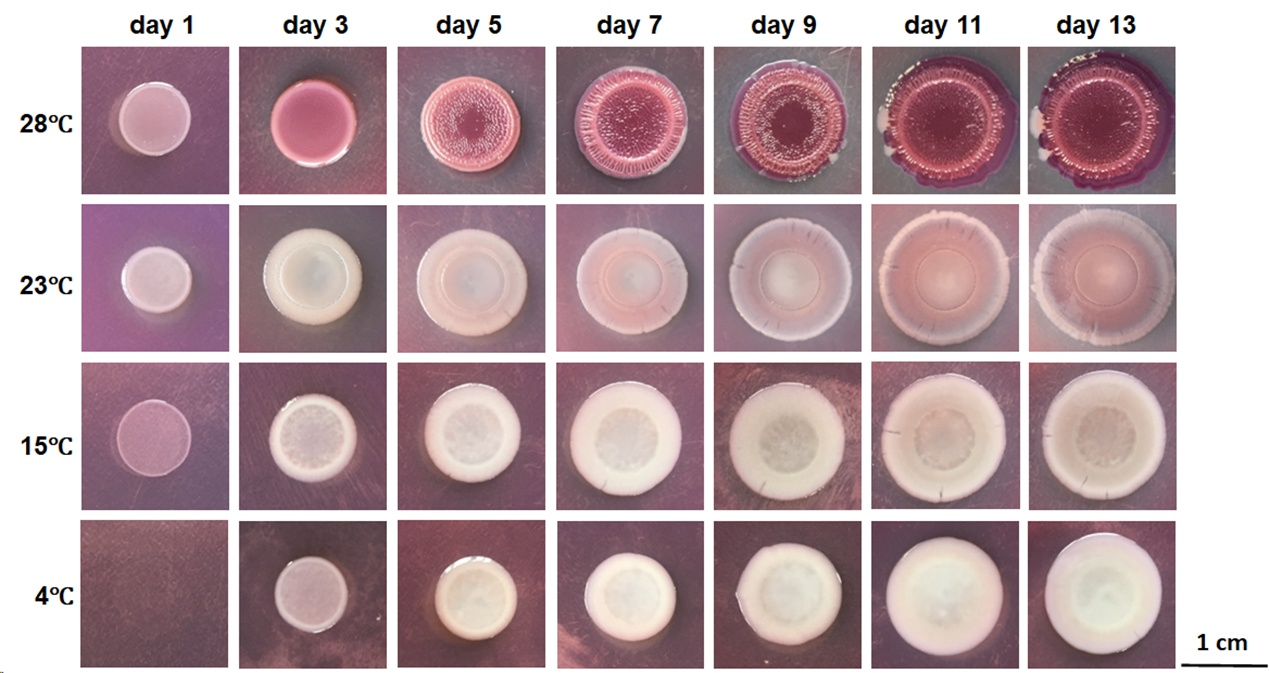
**

**Supplementary Fig. S1** Macrocolony morphology of UK4 cultivated on Congo red plates at 4°C, 15°C, 23°C, or 28°C for 13 days. Images from odd-numbered days are presented. The images are representative of a minimum of six biological replicates.

**
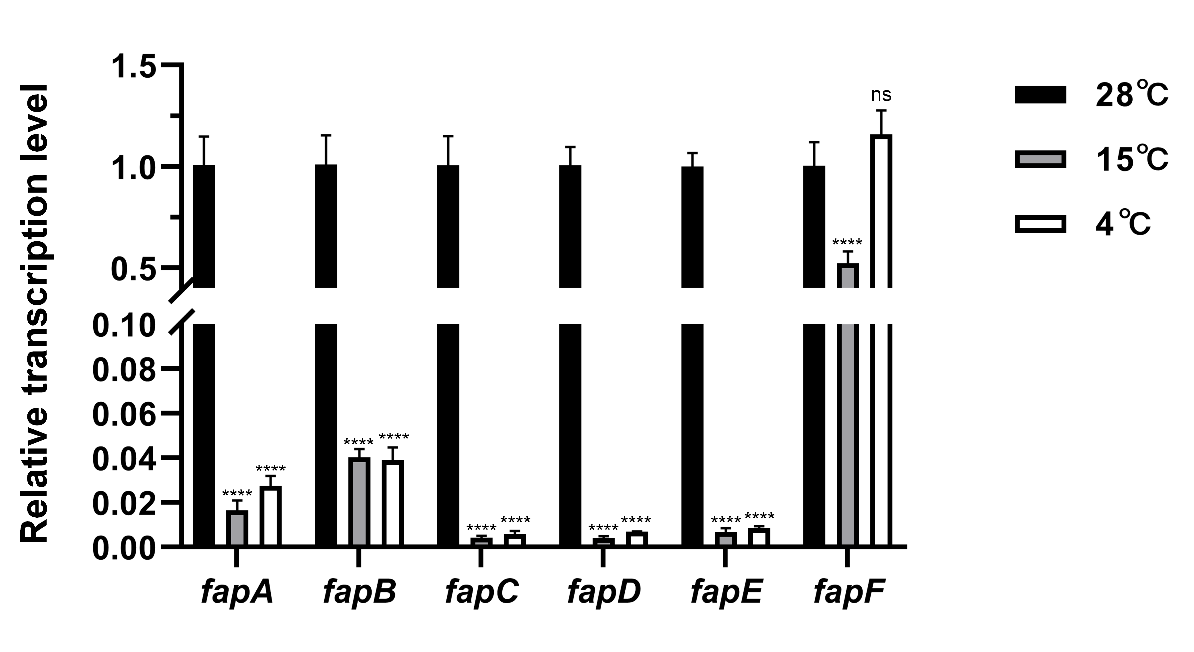
**

**Supplementary Fig. S2** Transcription levels of each gene in the *fapA–F* gene cluster determined via qRT-PCR after 3 days of incubation at 28°C, 15°C, and 4°C. The expression levels are relative to those at 28°C. Data are presented as mean ± SD of three biological replicates with three technical replicates each. Two-way ANOVA with Dunnett’s multiple comparisons test was used to determine statistical significance relative to the 28°C group (^****^*P* < 0.0001; ns, not significant).

**
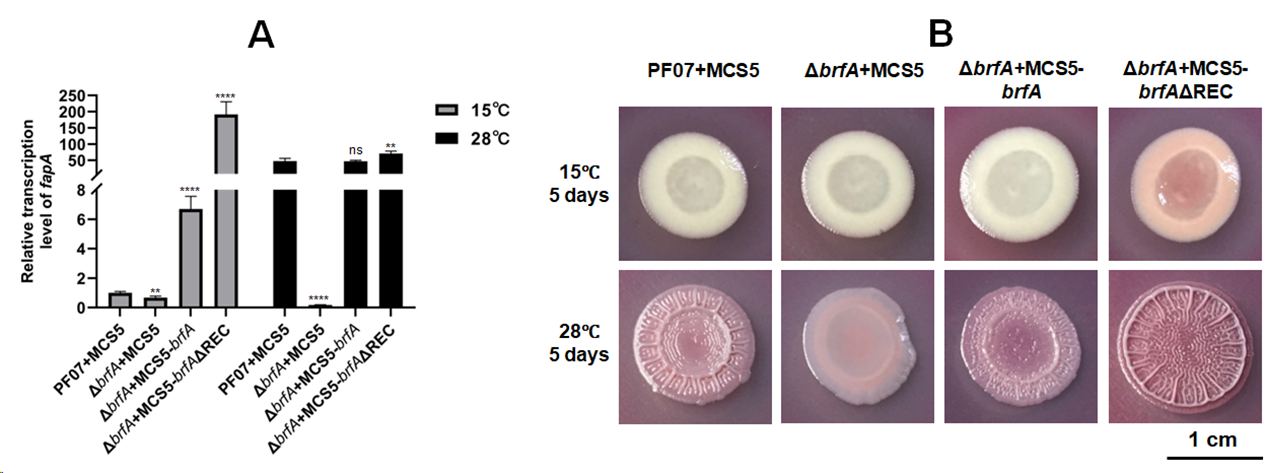
**

**Supplementary Fig. S3** Effects of *brfA* deletion and hyperactivation on *fapA* expression and macrocolony biofilm formation in PF07 at 28°C and 15°C. (A) Relative transcription levels of *fapA* in 2-day-old macrocolonies of wild-type PF07 (PF07+MCS5), a *brfA* mutant (Δ*brfA*+MCS5), the *brfA* mutant complemented with full-length *brfA* (Δ*brfA*+MCS5-*brfA*), and the *brfA* mutant complemented with truncated *brfA* (Δ*brfA*+MCS5-*brfA*ΔREC) determined by qRT-PCR. Results from our recent study indicated that removal of the N-terminal REC domain leads to the hyperactivation of BrfA (20). Data are presented as mean ± SD of three biological replicates with three technical replicates each. Two-way ANOVA was used to determine statistical significance relative to PF07+MCS5, followed by Dunnett’s multiple comparisons test (^**^*P* < 0.01, ^****^*P* < 0.0001; ns, not significant). (B) Macrocolony biofilms of the indicated strains formed on Congo red plates (scale bar = 1 cm). Representative images from at least three biological replicates are shown.


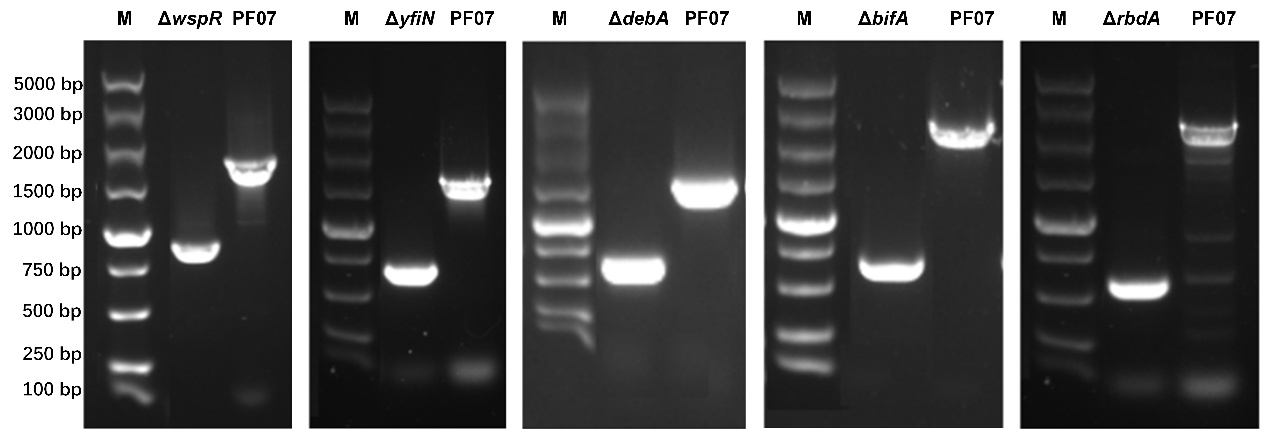


**Supplementary Fig. S4** PCR confirmation of the deletion mutants Δ*wspR*, Δ*yfiA*, Δ*debA*, Δ*bifA*, and Δ*rbdA*. The primer pairs *wspR*-TF/TR, *yfiA*-TF/TR, *debA*-TF/TR, *bifA*-TF/TR, and *rbdA*-TF/TR were used for mutant confirmation. M, DL5000 DNA ladder (Takara, China).


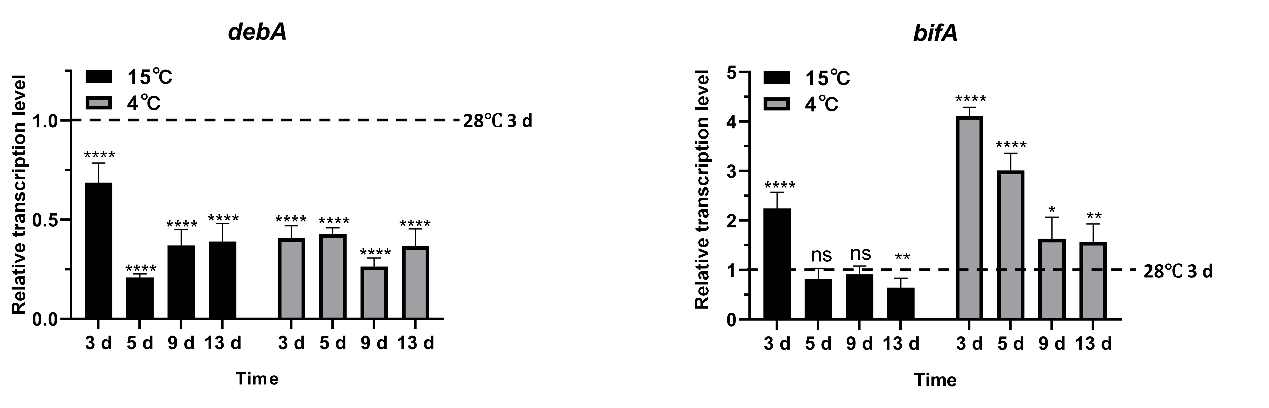


**Supplementary Fig. S5** Time-course analysis of *debA* and *bifA* expression in *P*. *fluorescens* PF07 macrocolonies at different temperatures, as determined by qRT-PCR. Expression levels are relative to those at 28°C for 3 days. Data are the mean ± SD of three biological replicates with three technical replicates each. One-way ANOVA with Dunnett’s T3 multiple comparisons test was used to determine statistical significance compared to the levels at 28°C for 3 days (^*^*P* < 0.05, ^**^*P* < 0.01, ^****^*P* < 0.0001; ns, not significant).

**
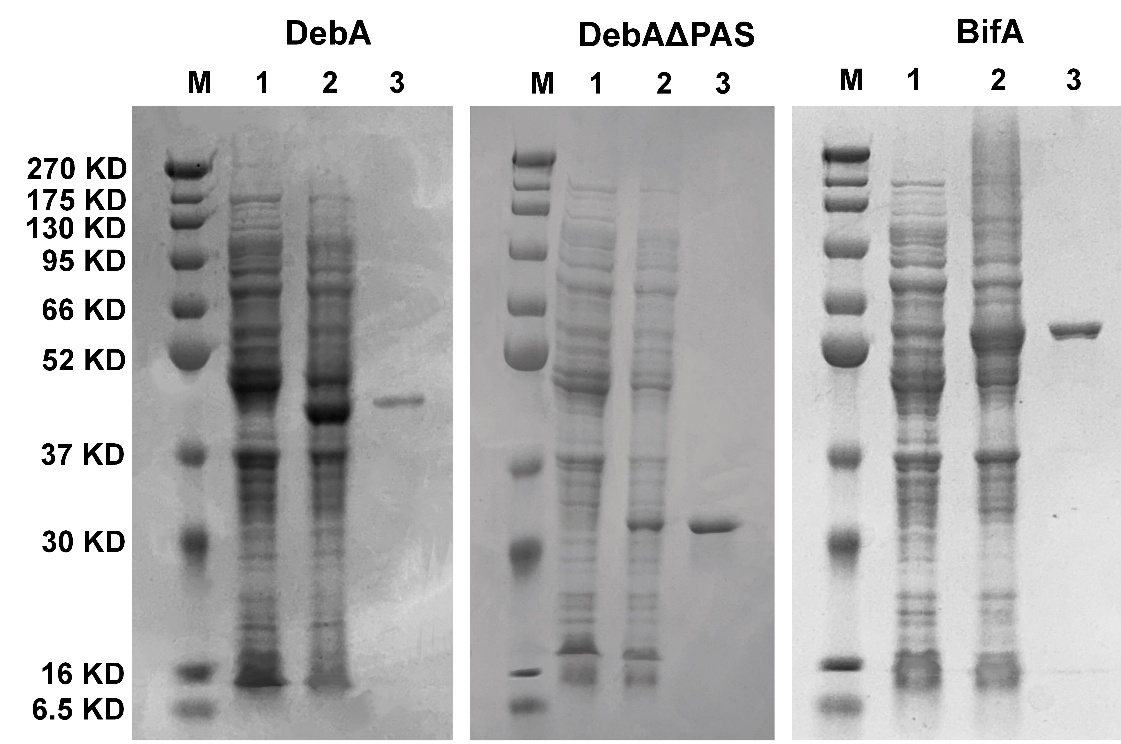
**

**Supplementary Fig. S6** Coomassie-stained gel of expressed and purified His-tagged DebA, DebAΔPAS, and BifA in *E. coli* BL21(DE3). M, protein molecular weight marker (Beyotime, China). Lane 1, soluble total proteins of *E. coli* BL21(DE3) harboring the empty vector following IPTG induction. Lane 2, soluble total proteins of *E. coli* BL21(DE3) expressing the target proteins. Lane 3, purified target proteins.

**Table S1 Primers used in this study.**

| **Primers** | **Primer sequences (5’- 3’)^a^** |
| --- | --- |
| **For qRT-PCR^b^** | |
| *fapA*-F  *fapA*-R | CAGATTCTACGCTCGACGACTAA  GATCGAGTGATGCTGGAACCA |
| *fapB*-F  *fapB*-R | GTGGCCAATCCACTGACGTACT  CCAGTACACCGTTGCCATTGCT |
| *fapC*-F  *fapC*-R | GCGATCGTAGACGTCGAGCAA  GCCGAGTTGTTCAGCGAAGCAT |
| *fapD*-F  *fapD*-R | GGGCTACTGGCTGGATGTTG  GCGCTTCATGTCCAGCATGGA |
| *fapE*-F  *fapE*-R | GCGGATTGTCAGCTTCGGT  GCCGGTAGTCTGCACATAGA |
| *fapF*-F  *fapF*-R | CCTTGAACGAGCGTATGAGTATG  CGTCACTGGACACAACCGACT |
| *fliC*-F  *fliC*-R | CGTTCAGCAACGTAGCGTTCCA  CGCGCTGATGTCGGTCATAC |
| *aprA*-F  *aprA*-R | GGCAGGATCGACTTGACCTACA  CCCAGGATTGCATGGCCAGAA |
| *debA*-F  *debA*-R | GCTACGATCACCATTCCCTGGAA  CGACAGCGGTATTCGGCTTGA |
| *bifA*-F  *bifA*-R | GCCAGTGTCGACAGCGAGAT  GCATAGCTGATCTGCGGTTGGT |
| 16S-F  16S-R | GGTGCCTTCGGGAACATTGAGAC  GTCTCCTTAGAGTGCCCACCATTAC |
| **For gene expression in pMMB206Gm** | |
| Gm-3 | GGATATTACGGCCTTtttaaaGACGCACACCGTGGAAA |
| Gm-4 | TCCATATTGGCCACGtttaaaGCGGCGTTGTGACAATTT |
| *debA*-A | TATGACCATGATTACgaattcCCACGGAGTATCGGCGTTA |
| *debA*-B | TCGACGGATCCCCGGgaattcTTATTGCGAAACCCGGTCCT |
| *wspR*-A | TATGACCATGATTACgaattcCACGATTGCTGGAGGTATTCG |
| *wspR*-B | TCGACGGATCCCCGGgaattcCTATTCGATCCCCACCTGGT |
| *yfiN*-A | TATGACCATGATTACgaattcTCAGTGATGAGCAAGTCTCGT |
| *yfiN*-B | TCGACGGATCCCCGGgaattcACAAGGGATCAACTCCTGTGT |
| *bifA*-A | TATGACCATGATTACgaattcTGACTGCCACCACGGGATT |
| *bifA*-B | TCGACGGATCCCCGGgaattcCTTTACAGGATGGCGGCGTT |
| *rbdA*-A | TATGACCATGATTACgaattcCTGGAGGGATCGTGTTTGGC |
| *rbdA*-B | TCGACGGATCCCCGGgaattcATGTTTCAGCGGAACGTGCC |
| **For gene deletion** |  |
| *debA*-MF1 | GAATTCGAGCTCGGTACCCCAACGCCTTGCTCGCCTATAT |
| *debA*-MR1 | TCAGGTCGGCGGGTGATTCCAAATCGGATGTATCGCCCAT |
| *debA*-MF2 | ATGGGCGATACATCCGATTTGGAATCACCCGCCGACCTGA |
| *debA*-MR2 | TCGACTCTAGAGGATCCCCCAACACGGAGAGCCTTGCAT |
| *debA*-TF | TGGTGCCGTATATCCCGGAA |
| *debA*-TR | AGGATTTGAAGCTGCGCAAG |
| *wspR*-MF1 | GAATTCGAGCTCGGTACCCGGTGCAGCGCTGTGCCGAGCTGA |
| *wspR*-MR1 | TTGGCGGTGTCCTGCAGGTTGCGGTCACCTGAATTACTAC |
| *wspR*-MF2 | GTAATTCAGGTGACCGCAACCTGCAGGACACCGCCAACCC |
| *wspR*-MR2 | TCGACTCTAGAGGATCCCCCCACGGTGTTGGTCGGTACGTGA |
| *wspR*-TF | GCGATGCCGTCGGCGTATTACTG |
| *wspR*-TR | TTCAAGCTTGGCGAGGTTGCCCT |
| *yfiN*-MF1 | GAATTCGAGCTCGGTACCCTGCTGCCCAGCCGTCTCAATGAT |
| *yfiN*-MR1 | TTGAACATTGGCGGCCGATTTCATGGCTCCGGCGCCCGAC |
| *yfiN*-MF2 | GGCGCCGGAGCCATGAAATCGGCCGCCAATGTTCAACACA |
| *yfiN*-MR2 | TCGACTCTAGAGGATCCCCCTGCTGTACCTGGCCAATGTCGT |
| *yfiN*-TF | GGCCCGACCCAATACACCGAC |
| *yfiN*-TR | GCCAAACGCCCAACCTTCGTC |
| *bifA*-MF1 | GAATTCGAGCTCGGTACCCCGAAGGCAAGACCCTGGAAG |
| *bifA*-MR1 | CTTTACAGGATGGCGGCGTTGAGCAACTTCACCGACAAGC |
| *bifA*-MF2 | CTTGTCGGTGAAGTTGCTCAACGCCGCCATCCTGTAAAG |
| *bifA*-MR2 | TCGACTCTAGAGGATCCCCTTCACCCAGGGACAGGCTG |
| *bifA*-TF | GGCAAGTCAGGTGAAGACGA |
| *bifA*-TR | CGGCGAGAGAAATGGCGATG |
| *rbdA*-MF1 | GAATTCGAGCTCGGTACCCCCAGATGCTCGCGGTGTTCAGCT |
| *rbdA*-MR1 | TACAGACCAGCAGATGTTGAGATCCCTTGCATCAGCACAG |
| *rbdA*-MF2 | GCTGATGCAAGGGATCTCAACATCTGCTGGTCTGTACACA |
| *rbdA*-MR2 | TCGACTCTAGAGGATCCCCGCCTGGGTTGCGCGACCTTGCTG |
| *rbdA*-TF | CAGGCTGATAGGGTATTGGTGTT |
| *rbdA*-TR | CTGTTCCACCACTGGCCAGCCAC |
| **For gene expression in pBBR1MCS-5** | |
| *debA*-MCS-A | GTCGACGGTATCGATaagcttTACTCGTTCGTCTACCAGGTG |
| *debA*-MCS-B | CAGGAATTCGATATCaagcttTTACTGCGAAACCCGGTCCT |
| **For construction of transcriptional and translational fusion reporter vectors** | |
| pHM25-1 | CACaagcttTCCAGTCGGGAAACCTGTC |
| pHM25-2 | CACaagcttATGACCATGATTACGGATTCACTG |
| *debA*-1 | GGTTTCCCGACTGGAaagcttTTCGTCTACCAGGTGCTGAT |
| Z*debA*-2 | TCTCCTTAGTCATCTaagcttCAAATCGGATGTATCGCCCAT |
| F*debA*-2 | CGTAATCATGGTCATaagcttCAAATCGGATGTATCGCCCAT |
| *bifA*-1 | GGTTTCCCGACTGGAaagcttTAATGGAACTCGCCAAGGGC |
| Z*bifA*-2 | TCTCCTTAGTCATCTaagcttGAGCAACTTCACCGACAAGC |
| F*bifA*-2 | CGTAATCATGGTCATaagcttGAGCAACTTCACCGACAAGC |
| **For expression and purification of recombinant proteins** | |
| *debA*e-1 | GTGCCGCGCGGCAGCcatatgGGCGATACATCCGATTTGG |
| *debA*e-2 | GTGCCGCGCGGCAGCcatatgAAACGCTCCCTGGAACAGC |
| *debA*e-3 | CTCGAGTGCGGCCGCaagcttTTACTGCGAAACCCGGTCC |
| *bifA*e-1 | GTGCCGCGCGGCAGCcatatgCTGCTGACCAAGCCCCTG |
| *bifA*e-2 | CTCGAGTGCGGCCGCaagcttTTACAGGATGGCGGCGTTATT |

^a^The restriction site regions of the primers are indicated in lower case. The sequences for recombination ligation of Exnase II in ClonExpress II One Step Cloning Kit are indicated by underlining.

^b^Primers *fapA-*F/R and 16S-F/R were from our previous report (19), and the other qRT-PCR primers were designed in this work.
